# Hybrid Ant-Baby Optimizer and BiLSTM framework for high-performance IoT intrusion detection

**DOI:** 10.3389/frai.2026.1795030

**Published:** 2026-05-07

**Authors:** Anbarasu Balakrishnan, Praveen Kumar Reddy Maddikunta

**Affiliations:** School of Computer Science Engineering and Information Systems, Vellore Institute of Technology, Vellore, India

**Keywords:** Ant-Baby Optimizer, bidirectional long short-term memory, CICIoT2023 dataset, class imbalance, feature selection, intrusion detection system, IoT security, metaheuristic optimization

## Abstract

**Introduction:**

Intrusion detection in Internet of Things networks continuously encounters issues with high-dimensional traffic data, class imbalance, and patterns of attacks.

**Methods:**

This paper proposes a hybrid intrusion detection framework which combines a novel bio-inspired Ant-Baby Optimizer for feature selection with a Bidirectional Long Short-Term Memory neural network for classification. The Ant-Baby Optimizer algorithm selects small, informative subsets of features using mutual information to replace features guaranteed to be low-relevance, thereby enhancing computational efficiency and interpretability. The selected features are re-shaped into pseudo-sequential order to allow, via the Bidirectional Long Short-Term Memory, to capture temporal dependencies in both directions.

**Results:**

The results on the CICIoT2023 dataset demonstrate superior detection accuracy (97.32%) balanced per-class precision, recall, and F1-scores (macro F1 = 0.9732), with an inference latency of 0.219 ms per sample and process time of over 4,500 samples per second.

**Discussion:**

Experiments and verification against state-of-the-art metaheuristic and deep learning approaches support the proposed framework as effective. The interdependence of computational complexity and dataset limitation cannot be overstated. In fact, these four qualities of performance, efficiency, interpretability, and adaptiveness, when considered as one, will pave the way for the proposed framework to perform at a high level in the real-world IoT intrusion detection systems. Moreover, future works will involve cross-domain validation, attention-based mechanisms, and online learning for zero-day attack handling.

## 1 Introduction

Intrusion detection remains one of the essential elements that helps to protect systems/networks against various cyber-attacks in the digital world that is changing very fast. Traditional intrusion detection systems are often unable to manage the enormous and high-dimensional data they receive, which leads to their detection accuracy going down and the number of false alarms going up. The function of feature selection in this context is not only to reduce the amount of calculations that have to be done but also to increase the accuracy of the detection (Kareem et al., 2022). For this reason, hybrid metaheuristic optimization has become the most favorable solution that a company can use to achieve the highest ability of the search by combining the different search paradigms used.

There is an increasing trend in the use of metaheuristic algorithms for feature subset selection. (Almomani et al. 2019) presented a core study, which covered the explorations of the metaheuristic techniques for locating the discriminative features for intrusion detection and creating the adaptable framework, which is always the basis for the current research trajectory. (Kunhare et al. 2022) moved the research area forward through mixing hybrid classifiers with metaheuristic-guided genetic algorithms and thereby accomplishing the detection performances with the highest merit by the simultaneous optimization of feature selection and classification. Correspondingly, (Alkanhel et al. 2023) constructed an advanced hybrid metaheuristic optimization model and demonstrated that IDS performance could be significantly improved by the synergistic effect of the different layers of search strategies.

While networks become more and more complex, the investment in Big Data is the foremost barrier against the feature selection efforts that are not scalable and less robust. (Kumar and Mohan 2021) have resolved the problem by the creation of a hybrid metaheuristic optimization approach that is targeted at the data environment of large-scale, which not only characterizes the classification model but also brings about the optimization of the model through the selection of an intelligent feature subset. The work of (ElDahshan et al. 2022) is followed by the design of an intrusion detection system across different levels, governed by metaheuristic optimization, thus strengthening the argument in favor of a multi-tier approach to a complex detection landscape.

The complexity of current network attacks has raised the demand for detection frameworks with the ability to classify flexibly and distinguish multiple classes. The paper of (Akinola et al. 2022) deals with a comprehensive review of the multiclass feature selection through the application of metaheuristic algorithms, emphasizing the need for the versatility and adaptability of algorithms when faced with the real world scenarios that contain heterogeneous intrusion classes.

The IoT situation complicates matters further. To address the issue, (Jing et al. 2025) have recently presented a method based on hierarchical arithmetic optimization for intrusion detection in the IoT, which is a step in the direction of low-latency solutions that are aware of the resource. Their work emphasize the necessity of hierachical lightweight metaheuristic schemes that are able to efficiently operate in tightly resource environments, which is a widely unexplored field of research.

On the other hand, the multi-objective feature selection paradigm is still very powerful. (Ghanem et al. 2020) proposed a multi-objective packing method combined with neural network classification that simultaneously optimizes multiple criteria, thereby providing an intelligent model for future systems that need to make tradeoffs between recognition accuracy, speed, and resource utilization.

Collectively, the above authorities reveal a clear strategic continuum:

- Early metaheuristic-only frameworks established core feasibility (Almomani et al., 2019).
- Hybrid models have combined search heuristics with classification optimization (Kunhare et al., 2022; Alkanhel et al., 2023).
- Specialized strategies have addressed the scale and structure of big data and hierarchical systems (Kumar and Mohan, 2021; ElDahshan et al., 2022).
- The multi-class and multi-criteria approach increases the flexibility of the algorithm (Akinola et al., 2022; Ghanem et al., 2020).
- IoT-centric frameworks highlighted the use of lightweight, hierarchical metaheuristic structures (Jing et al., 2025).

As a result, the present study introduces a future-oriented hybrid metaheuristic optimization framework that considers the trade-off between detection performance, computational efficiency, and adaptability to different deployment contexts such as IoT and large-scale network environments. The proposed method, which results in higher detection efficacy while limiting resource wastage, achieves this by the integration of hierarchical search strategies, multi-objective optimization, and classifier synergy.

By Summarization, the key contributions of this work are:

1. A **hybrid metaheuristic architecture** which combines compatible search mechanisms for stable feature subset selection.
2. A **hierarchical design** Designed for scalable performance in high-bandwidth networks and resource-constrained IoT systems.
3. A **multi-objective optimization scheme** This harmoniously combines detection accuracy, execution efficiency and compactness for all functions.
4. **Rigorous validation** showcase notable improvements in detection rate and false-positive reduction over previous models.

Through this conjoint effort, we are not only resolving the issues demanded by the present context but also, with strategic agility, foreseeing and preparing for the ever-changing threat scenario.

## 2 Related work

Feature selection plays a significant role in the traffic of a network intrusion detection system (IDS). First, it reduces the dimensionality of the large volume of traffic data. Second, it improves the classification accuracy, and third, it reduces the computational cost. Over the last decade several researchers used meta-heuristic algorithms to solve this challenge. In most cases, they combine these algorithms with hybrid classification systems to keep up with the continuously increasing complexity of cyber-attacks.

### 2.1 Metaheuristic-based feature selection

(Khorram and Baykan 2018) used metaheuristic algorithms for feature selection in network intrusion detection and showed that the optimal subsets could lead the detection rates to be greatly increased as compared to statistical methods of a standard nature. (Kareem et al. 2022) have taken this method a step further into the domain of IoT, by developing a hybrid metaheuristic feature selection model which is able to simultaneously optimize detection accuracy and computational efficiency. (Shanbhag et al. 2024) in the same way, fused metaheuristics with machine learning classifiers to identify the network packets that are malicious and they have been able to facilitate a significant increase in recall for the categories of the rare kind of attacks.

(Gong et al. 2025) came up with a new metaheuristic optimization technique of feature selection in IDS. Their approach was able to outperform the conventional heuristic search methods in terms of speeding up the convergence of the optimal feature subsets. (Ghanbarzadeh et al. 2023) developed an IDS that is metaheuristic-optimized and that has demonstrated a high level of accuracy while keeping the false-positive rate low, especially in the case of datasets that are imbalanced.

### 2.2 Advanced and enhanced metaheuristics

(Yu et al. 2025) engineered a smart heuristic algorithm for feature selection in IDS that included adaptive strategies to enhance the exploration-exploitation balance. (Agrawal et al. 2021) surveyed the use of metaheuristics in feature selection over the last decade, and they pointed out that there is a trend of shifting toward hybrid models and ensemble techniques. Ensemble strategy (Thockchom et al., 2023) by them provides a very good balance of detection power and resistance to fluctuations of the recent network traffic, thus, attackers are definitely kept in the dark.

(Almomani 2021) developed a hybrid bio-inspired metaheuristic model for an IDS that was able to achieve higher detection accuracy and increased robustness against various attack types than a heuristic-alone technique. (Velliangiri and Karthikeyan 2020) introduced a hybrid optimization method that could effectively trade off feature relevance and redundancy, thereby classifier stability was enhanced.

### 2.3 Swarm intelligence and evolutionary approaches

(Kunhare et al. 2020) used particle swarm optimization (PSO) for feature selection and, as a result, they were able to reduce the number of features significantly while maintaining the performance of the detection accuracy. Similarly, Ant Colony Optimization (ACO) and Genetic Algorithms (GA) have been widely adopted due to their global search capabilities. However, these methods often rely on computationally expensive operations such as crossover (in GA), velocity updates (in PSO), or pheromone tracking (in ACO), which can limit scalability in large-scale or real-time environments. (Hossain and Islam 2024) came up with a hybrid feature-selection method along with an ensemble-based classifier which, by a large margin, increased the identification of DDoS attacks and thus, made a step toward more stable cybersecurity solutions. Prior to this several foundational works have also been instrumental in helping the research and development of this field.

(Alkanhel et al. 2023) considered hybrid metaheuristic optimization for the network intrusion detection system, while (Almomani et al. 2019) examined metaheuristic frameworks as a potential solution for feature selection that can be generalized. The integration of hybrid metaheuristic-classifier was further refined by (Kumar and Mohan 2021), (ElDahshan et al. 2022), and (Akinola et al. 2022) respectively, where they addressed issues such as scalability, hierarchical structure, and multiclass classification. The development of IoT-specific solutions have emerged as a key area of interest. (Jing et al. 2025) introduced a hierarchical arithmetic optimization strategy for intrusion detection in IoT, while (Kareem et al. 2022) delivered a study that emphasized the production of light solutions but kept their accuracy intact for devices with limited resources.

### 2.4 Summary of Related Work

Table 1 summarizes the key insights from the reviewed literature, along with the metaheuristic method, dataset, and the primary reported outcomes.

**Table 1:** Summary of related work on metaheuristic-based feature selection for IDS.

Author(s)/year	Metaheuristic method	Domain/dataset	Key outcomes
(Khorram and Baykan 2018)	Generic metaheuristics	NSL-KDD	Improved detection accuracy, reduced computation time
(Kareem et al. 2022)	Hybrid metaheuristics	IoT traffic datasets	High accuracy with low computational load
(Shanbhag et al. 2024)	Hybrid metaheuristics + ML classifiers	CICIDS2017	High recall for rare attacks, balanced precision
(Gong et al. 2025)	New metaheuristic design	KDD Cup 99	Faster convergence to optimal feature subset
(Ghanbarzadeh et al. 2023)	Metaheuristic optimization	UNSW-NB15	High accuracy, low false positives
(Yu et al. 2025)	Enhanced heuristic optimizer	NSL-KDD, CICIDS2017	Better exploration-exploitation trade-off
(Agrawal et al. 2021)	Survey of metaheuristics	Multiple datasets	Trend toward hybrid/ensemble solutions
(Thockchom et al. 2023)	Ensemble Learning-Based NIDS	Benchmark IDS Datasets (e.g., NSL-KDD, CIC-IDS2017)	High accuracy with improved generalization
(Almomani 2021)	Bio-inspired hybrid metaheuristics	NSL-KDD	Superior robustness vs. standalone methods
(Velliangiri and Karthikeyan 2020)	Hybrid optimization	KDD Cup 99	Improved stability, balanced feature relevance
(Kunhare et al. 2020)	Particle Swarm Optimization	NSL-KDD	Dimensionality reduction with maintained accuracy

### 2.5 Insights and research gap

It reveals that the metaheuristic algorithms with hybrid and adaptive features are way better than single-strategy algorithms in the selection of features for IDS. The use of multi-objective optimization, like in (Ghanem et al. 2020); (Yu et al. 2025), is thus very helpful in achieving a good balance between accuracy and efficiency. Besides, the specificity of the domain, e.g., IoT-oriented optimization in (Jing et al. 2025); (Kareem et al. 2022), gives performance advantages that are specifically directed at the limited-resource type of environments.

Despite these advancements, several critical gaps remain:

- **Computational complexity:** many existing methods rely on expensive operations such as crossover, velocity updates, and pheromone tracking, limiting scalability.
- **Lack of lightweight optimization:** few approaches focus on simple yet effective feature selection mechanisms that are computationally efficient and interpretable.
- **Limited modeling of feature dependencies:** traditional classifiers often fail to capture complex inter-feature relationships in high-dimensional intrusion data.
- **Generalization challenges:** many models are evaluated on specific datasets, with limited validation across diverse network environments.

To address these gaps, the present work proposes a lightweight Ant-Baby Optimizer (ABO) that eliminates complex evolutionary operations through a mutual-information-guided mutation strategy. Furthermore, a BiLSTM-based classifier is employed to model bidirectional dependencies among selected features, enabling improved detection of subtle and multi-class intrusion patterns. Figuring out these aspects can, therefore, be a way of speeding up the metaheuristic-based IDS journey from being mere research prototypes to production-grade systems that are mission-critical.

## 3 Ant-Baby Optimizer (ABO) framework

### 3.1 Overview

A lightweight bio-inspired feature selection algorithm named as Ant-Baby Optimizer that operates by the principle of mutation - based reproduction observed in nature. Unlike traditional evolutionary and swarm-based optimization methods such as Genetic Algorithms (GA), Particle Swarm Optimization (PSO), and Ant Colony Optimization (ACO), the proposed ABO eliminates computationally expensive operations such as crossover, velocity updates, and pheromone tracking. Instead, ABO employs a simplified yet effective mutation-driven strategy, where less informative features are iteratively replaced by more relevant ones based on mutual information (MI) scores. This design significantly reduces computational complexity while maintaining strong feature selection capability.

### 3.2 Problem definition

Let the dataset be represented as:


$$\begin{array}{l} X\in {\mathbb{R}}^{N\times M},\;y\in {\mathbb{R}}^{N} \end{array}$$ (1)

where:

- *N* = number of samples
- *M* = total number of features
- *y* = target class labels

The goal is to select an optimal subset of *k* features:


$$\begin{array}{l} S^{*}=\arg\underset{S\subset \left\{1,2,\ldots ,M\right\},|S|=k}{\max}F\left(S\right) \end{array}$$ (2)

where *F*(*S*) denotes the fitness function.

### 3.3 Fitness Function

The mutual information score for feature *f*_*i*_ with respect to the target *y* is defined as:


$$\begin{array}{l} \text{MI}\left(f_{i},y\right)=\underset{f_{i},y}{\sum }p\left(f_{i},y\right)\log\left(\frac{p\left(f_{i},y\right)}{p\left(f_{i}\right)p\left(y\right)}\right) \end{array}$$ (3)

where *p*(·) denotes the probability of mass function.

The fitness of a subset *S* is given by:


$$\begin{array}{l} F\left(S\right)=\underset{i\in S}{\sum }\text{MI}\left(f_{i},y\right) \end{array}$$ (4)

### 3.4 Initialization

A population of *n*_*a*_ ants (candidate feature subsets) is initialized:


$$\begin{array}{l} P_{0}=\left\{S_{1},S_{2},\ldots ,S_{n_{a}}\right\} \end{array}$$ (5)

where each *S*_*j*_ contains *k* features randomly selected from {1, …, *M*}.

### 3.5 Mutation mechanism

At each generation *t*, for the best-performing subset *S*_best_:

1. Randomly select a feature *f*_out_ ∈ *S*_best_ to remove.
2. Identify the best available feature *f*_in_ ∈ {1, …, *M*}\*S*_best_ such that:

$$\begin{array}{l} f_{\text{in}}=\arg\underset{j\notin S_{\text{best}}}{\max}\text{MI}\left(f_{j},y\right) \end{array}$$ (6)

3. Replace *f*_out_ with *f*_in_:

$$\begin{array}{l} S_{\text{new}}=\left(S_{\text{best}}\backslash \left\{f_{\text{out}}\right\}\right)\cup \left\{f_{\text{in}}\right\} \end{array}$$ (7)

This mutation strategy ensures monotonic improvement by consistently introducing highly informative features into the subset.

### 3.6 Selection

The new population *P*_*t*_ is formed by mutating *S*_best_ multiple times. The best subset for generation *t*+1 is:


$$\begin{array}{l} S_{\text{best}}^{\left(t+1\right)}=\arg\underset{S\in P_{t}}{\max}F\left(S\right) \end{array}$$ (8)

### 3.7 Termination

The process continues for *T*_max_ iterations or until:


$$\begin{array}{l} F\left(S_{\text{best}}^{\left(t+1\right)}\right)-F\left(S_{\text{best}}^{\left(t\right)}\right)<\epsilon \end{array}$$ (9)

where ϵ is a small threshold.

### 3.8 Output

The final selected subset


$$\begin{array}{l} S^{*}=S_{\text{best}}^{\left(T_{\max}\right)} \end{array}$$ (10)

This subset is subsequently used for training the classification model.

### 3.9 Algorithm summary

1. Compute MI(*f*_*i*_, *y*) using Equation 3 for all features *i* = 1, …, *M*.
2. Initialize *n*_*a*_ ants with random feature subsets of size *k* using Equation 5.
3. Evaluate fitness using Equation 4 for each ant *F*(*S*).
4. For *t* = 1 to *T*_max_:

- Using Equations 6, 7 mutate the best ant according to the mutation rule.
- Evaluate new subsets and update *S*_best_ as shown in Equations 8, 9.

5. Based on the outcome of Equation 10 return *S*_best_ as the optimal feature subset.

### 3.10 Key advantages of ABO

The proposed ABO offers several advantages over conventional metaheuristic approaches:

- **Computational efficiency:** eliminates expensive operations such as crossover and velocity updates.
- **Deterministic improvement:** uses mutual information to guide feature replacement, ensuring consistent fitness improvement.
- **Simplicity and interpretability:** provides a transparent feature selection mechanism compared to black-box evolutionary models.
- **Scalability:** suitable for high-dimensional datasets and resource-constrained environments such as IoT systems.

## 4 Proposed methodology

The proposed methodology synergistically integrates the bio-inspired feature selection algorithm, ABO, with a BiLSTM network specifically tailored for tabular IDS datasets. This hybridize approach aims to improve detection accuracy by efficiently identifying the most relevant features through the proposed ABO algorithm while simultaneously enabling deep representation learning through sequential modeling. BiLSTM's ability of temporal modeling capabilites is used to caputure the complex network patterns of the network traffic data. This enables to learn and recognize the complex attacks more effectively. The four primary phases of the methodology are feature selection, model construction, performance evaluation, and data preprocessing. In the process of generating high quality inputs to aid the models the dataset is cleaned first and followed by normalization and encoding in the preprocessing phase. The ABO algorithm then carries out an optimization-driven feature selection to improve computational efficiency, minimize redundancy, and reduce dimensionality without compromising predictive power. The same is illustrated in Algorithm 1.

Algorithm 1 **Proposed Hybrid Ant–Baby Optimizer.**

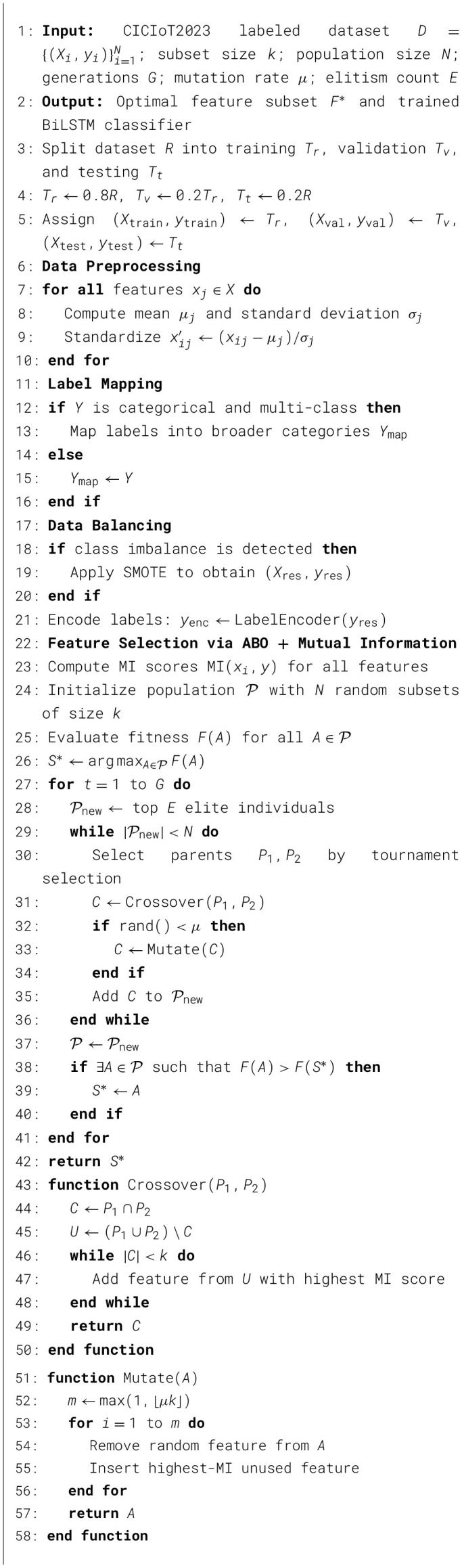

**Figure 1:**
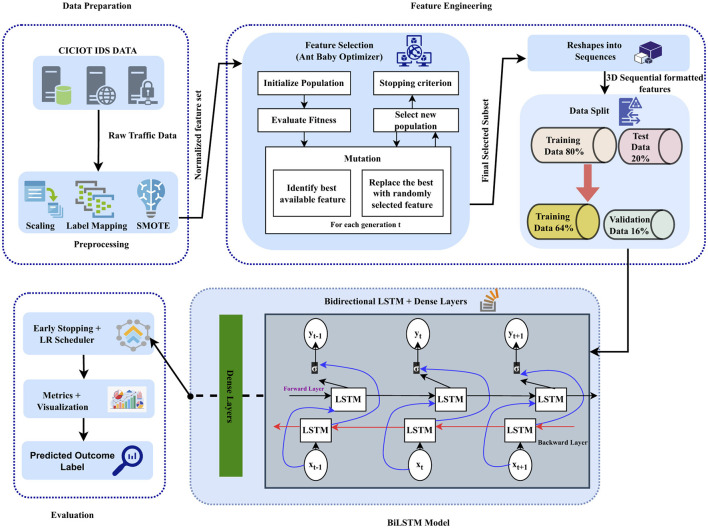
Architecture diagram of the proposed intrusion detection framework integrating Ant-Baby Optimizer (ABO) and BiLSTM network.

After selecting the required features the BiLSTM model process the features with its unique ability of working with both preceding and succeeding data points. This improves the detection of low profile intrusions patterns. At the final stage, several metrics are used to validate the frameworks trustworthiness. The Algorithm 2. briefly constructs the BiLSTM model and simulation in the real time of the model. Figure 1 depicts the workflow of the proposed system, offering clear visual representation of each stage and phases and their interconnections within the overall architecture. It presents the end-to-end workflow of the proposed system, offering a clear visual overview of each stage and their interrelations within the overall architecture.

Algorithm 2 **BiLSTM Model Construction and Evaluation**

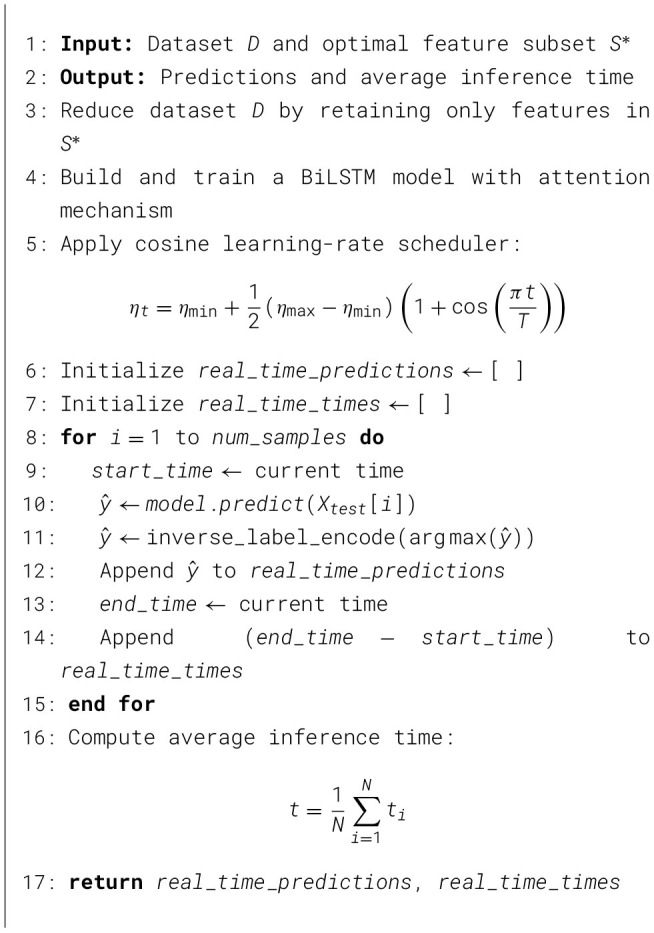

### 4.1 Dataset description

In this research, the *CICIoT2023* dataset used to identify and classify various attack types within the IoT environment. The efficiency of an IDS in correctly classifying and detecting malicious threats depends significantly on the robustness of the training data. It is used to optimize its identification models. Reliable threat assessment depends on high-quality datasets that thoroughly encompass the pertinent attack vectors (Neto et al., 2023). The *CICIoT2023* dataset, newly released by the Canadian Institute for Cybersecurity provides a resource for creating security analytics solutions tailored to realistic IoT environments.

**Figure 2:**
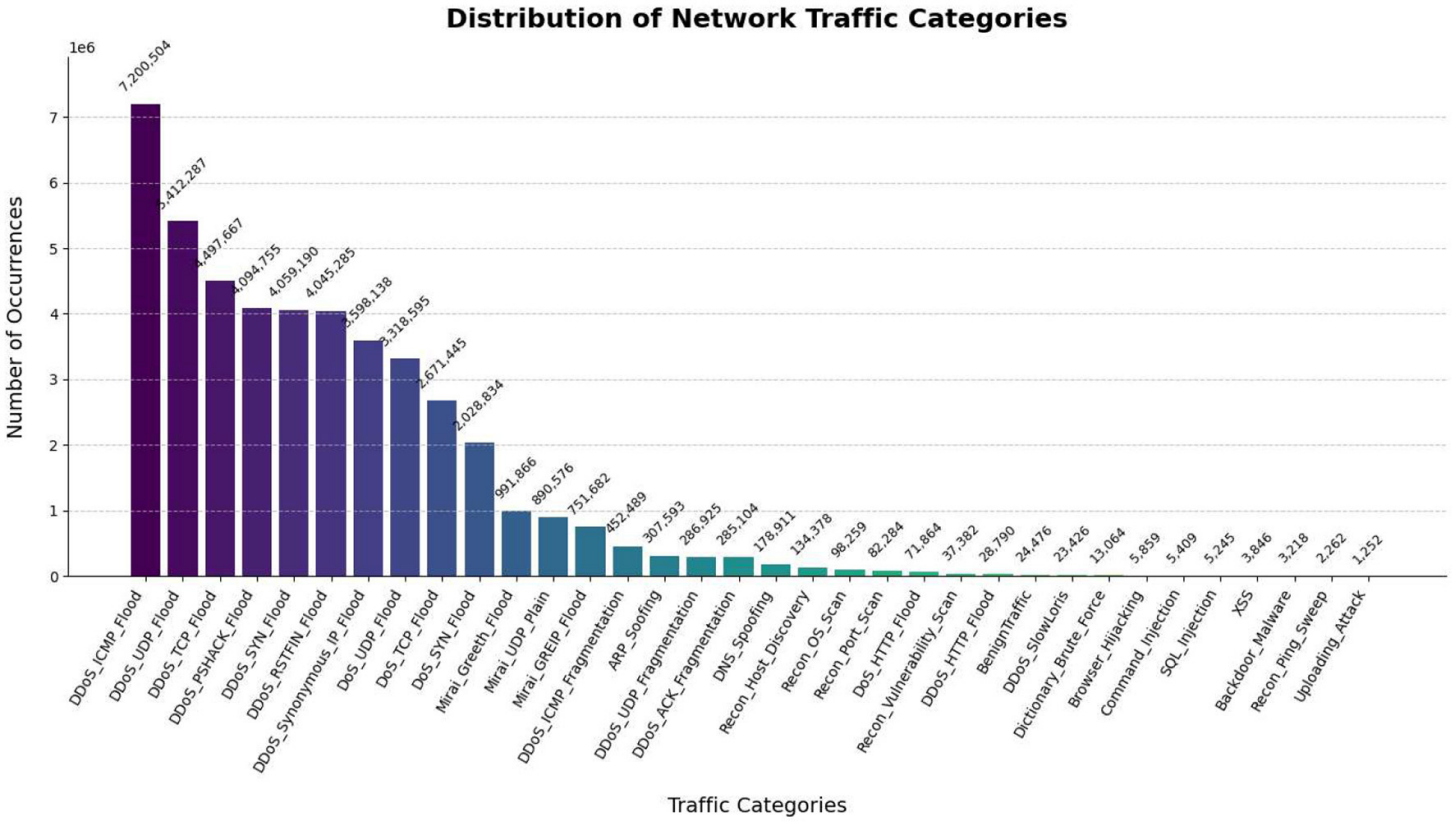
Distribution of attack types in the CICIoT2023 dataset.

This dataset stands out due, to its breadth and structure containing 33 attack varieties carried out on a simulated IoT network made up of 105 devices. The attacks are grouped into seven primary categories: Distributed Denial-of-Service (DDoS), Denial-of-Service (DoS), Reconnaissance, Web-based attacks, Brute-force attacks, Spoofing attacks, and Mirai attacks. Table 2 presents the distribution of attack instances, while Figure 2 illustrates the graphical representation of the data. The frequency and scope of various threats can be inferred from quantitative data on the number of attack instances for each sub-type. Granular analysis is made possible by the multi-tiered categorical system, which ranges from general classes like DDoS and Web-based attacks to particular attack techniques like SYN Flood and SQL Injection.

**Table 2:** Number of instances of attacks in CICIoT2023 dataset.

S.No	Category of attacks	Number of instances
1	**Distributed denial-of-service (DDoS)**	
	ACK fragmentation	285,104
	UDP flood	5,412,287
	SlowLoris	23,426
	ICMP flood	7,200,504
	RSTFIN flood	4,045,285
	PSHACK flood	4,094,755
	HTTP flood	28,790
	UDP fragmentation	286,925
	ICMP fragmentation	452,489
	TCP flood	4,497,667
	SYN flood	4,059,190
	Synonymous IP flood	3,598,138
2	**Denial-of-Service (DoS)**	
	TCP flood	2,671,445
	HTTP flood	71,864
	SYN flood	2,028,834
	UDP flood	3,318,595
3	**Reconnaissance**	
	Ping sweep	2,262
	OS scan	98,259
	Vulnerability scan	37,382
	Port scan	82,284
	Host discovery	134,378
4	**Web-based attacks**	
	SQL injection	5,245
	Command injection	5,409
	Backdoor malware	3,218
	Uploading attack	1,252
	XSS	3,846
	Browser hijacking	5,859
5	**Brute-force attacks**	Dictionary Brute Force: 13,064
6	**Spoofing attacks**	
	ARP spoofing	307,593
	DNS spoofing	178,911
7	**Mirai attacks**	
	GREIP flood	751,682
	Greeth flood	991,866
	UDP plain	890,576

Comparative analysis of the relative frequency and impact of various attack vectors is made easier by the thorough quantification. While the complete *CICIoT2023* dataset encompasses approximately 45.6 million traffic records, employing the entire dataset for model development is computationally prohibitive due to constraints in memory, processing time, and storage overhead. Training a deep sequential model such as BiLSTM on tens of millions of records would require distributed Graphical Processing Unit (GPU) clusters and extended runtime, which are not practical for the scope of this work. Furthermore, extremely large datasets often introduce redundancy, as dominant attack categories such as DDoS and DoS constitute most of the records, overshadowing minority classes. This imbalance can lead to biased classification.

### 4.2 Computational data reduction

A stratified subset of 1,048,576 records was extracted to address these issues and guarantee proportionate representation of each attack category. This strategy accomplishes several goals:

- **Computational efficiency:** without sacrificing experimental rigor, deep models like BiLSTM can be trained on single-GPU or workstation setups by reducing the dataset size by more than 97%.
- **Class balance maintenance:** by maintaining sufficient representation of minority attack categories, stratification reduces skewness and possible bias.
- **Redundancy elimination:** to avoid biasing the model and increasing training time, oversampled majority classes are subsampled.
- **Reproducibility and controlled experimentation:** iterative model evaluation, controlled hyperparameter tuning, and repeatable experiments are made easier by using a representative yet manageable subset.

Previous research has shown that statistically representative, carefully sampled subsets can maintain predictive performance on par with models trained on the entire dataset. As a result, the reduced and balanced subset offers a workable balance between *robust, generalisable model development* and *experimental tractability*, providing a strong basis for the suggested ABO–BiLSTM intrusion detection framework.

The most common attacks, according to data analysis, are DDoS and DoS, which flood targets with massive amounts of traffic coming from multiple or single sources, respectively. The dataset highlights the prevalence of these vectors with over 8 million DoS attacks and over 29 million DDoS attack instances. Attacks that are statistically less common but have a significant impact, like Mirai botnet attacks that target IoT devices, also require attention. Furthermore, web application attacks, spoofing, brute force, and reconnaissance continue to be major ongoing threats in significant quantities. The reduction stage is intended to facilitate controlled and reproducible experimentation under realistic resource constraints but not to claim universal generalization. The reduced dataset preserves the benchmark's multiclass structure and enables fair comparison among internally evaluated model variants. However, the findings of this study are limited to the evaluated benchmark setting and do not on their own establish cross-dataset robustness.

### 4.3 Data preprocessing

Several transformations are performed on the raw IoT intrusion dataset to prepare it for modeling:

- **Feature scaling:** standardScaler is used to normalize all numerical attributes to ensure zero mean and unit variance:

$$\begin{array}{l} x_{ij}^{\prime }=\frac{x_{ij}-\mu_{j}}{\sigma_{j}} \end{array}$$ (11)

Here, the mean and standard deviation of the *j*^*th*^ features are μ_*j*_ and σ_*j*_, respectively.

- **Label mapping:** Specific attack types are grouped into broader classes such as BruteForce, DoS, DDoS, Mirai, Recon, Spoofing, Web, and Benign to reduce class fragmentation.
- **Balancing:** To overcome class imbalance the dataset has to undergo resampling so that each class has an equal number of samples. This is achieved by downsampling majority classes and oversampling minority ones. To further enhance class representation, the Synthetic Minority Oversampling Technique (SMOTE) is applied, which generates new synthetic samples by interpolating between a data point *x*_*i*_ and one of its nearest neighbors *x*_*nn*_:

$$\begin{array}{l} x_{\text{new}}=x_{i}+\text{λ}\left(x_{nn}-x_{i}\right),\text{ λ}\sim U\left(0,1\right) \end{array}$$ (12)

This approach ensures a uniform class distribution and provides more diverse, realistic data points for model training.

### 4.4 Class distribution after balancing

Table 3 shows the initial class distribution after the labels were remapped using Equation 11. One can see that the dataset was extremely unbalanced, as most of the instances were from the DDoS class. In order to reduce the effect of class imbalance, fixed-size sampling was employed through Equation 12, which led to a balanced dataset with 50,000 samples per class, as illustrated by Table 4.

**Table 3:** Original class distribution after label remapping (dataset size: 1,048,575).

Class label	Samples	Percentage
DDoS	763,525	72.8%
DoS	181,481	17.3%
Mirai	59,233	5.6%
Benign	24,476	2.3%
Spoofing	11,053	1.0%
Recon	7,945	0.8%
Web	538	0.05%
BruteForce	324	0.03%

**Table 4:** Balanced class distribution after resampling (dataset size: 400,000).

Class label	Samples	Percentage
Benign	50,000	12.5%
BruteForce	50,000	12.5%
DDoS	50,000	12.5%
DoS	50,000	12.5%
Mirai	50,000	12.5%
Recon	50,000	12.5%
Spoofing	50,000	12.5%
Web	50,000	12.5%

### 4.5 Data splitting

To conduct a dependable assessment and avert overfitting, the dataset was divided into three separate subsets: 80% for training, 20% (taken from the training set) for validation, and 20% for testing. Stratified sampling was used to maintain the distribution of all attack categories in the splits. The training set served for model fitting and parameter optimization, whereas the validation set was used for hyperparameter tuning and early stopping. The performance measures that have been reported as the final evaluation in this paper are solely obtained from the test set that has never been seen before, thus providing an impartial evaluation of the proposed framework.

### 4.6 Feature selection using Ant-Baby Optimizer

Feature selection plays a critical role in IDS with respect to the high-dimensional IoT datasets where redundant and irrelevant features can significantly degrade model performance. The ABO is used to select the best subset of features that can discriminate the classes effectively while also reducing redundancy. This ensures that each iteration results in a monotonic or near-monotonic improvement in feature quality. The first step is to calculate the mutual information score MI(*f*_*i*_, *y*) for each feature *f*_*i*_, where this score measures the amount of dependence between the feature and the target class *y* and is computed using the Equation 3. Features having higher mutual information scores are regarded as being more useful for classification. The proposed ABO framework adopts a simplified yet effective deterministic optimization strategy also it eliminates computationally expensive mechanisms of the other optimization algorithms and instead employs a direct mutation-based improvement strategy guided by MI scores.

**Figure 3:**
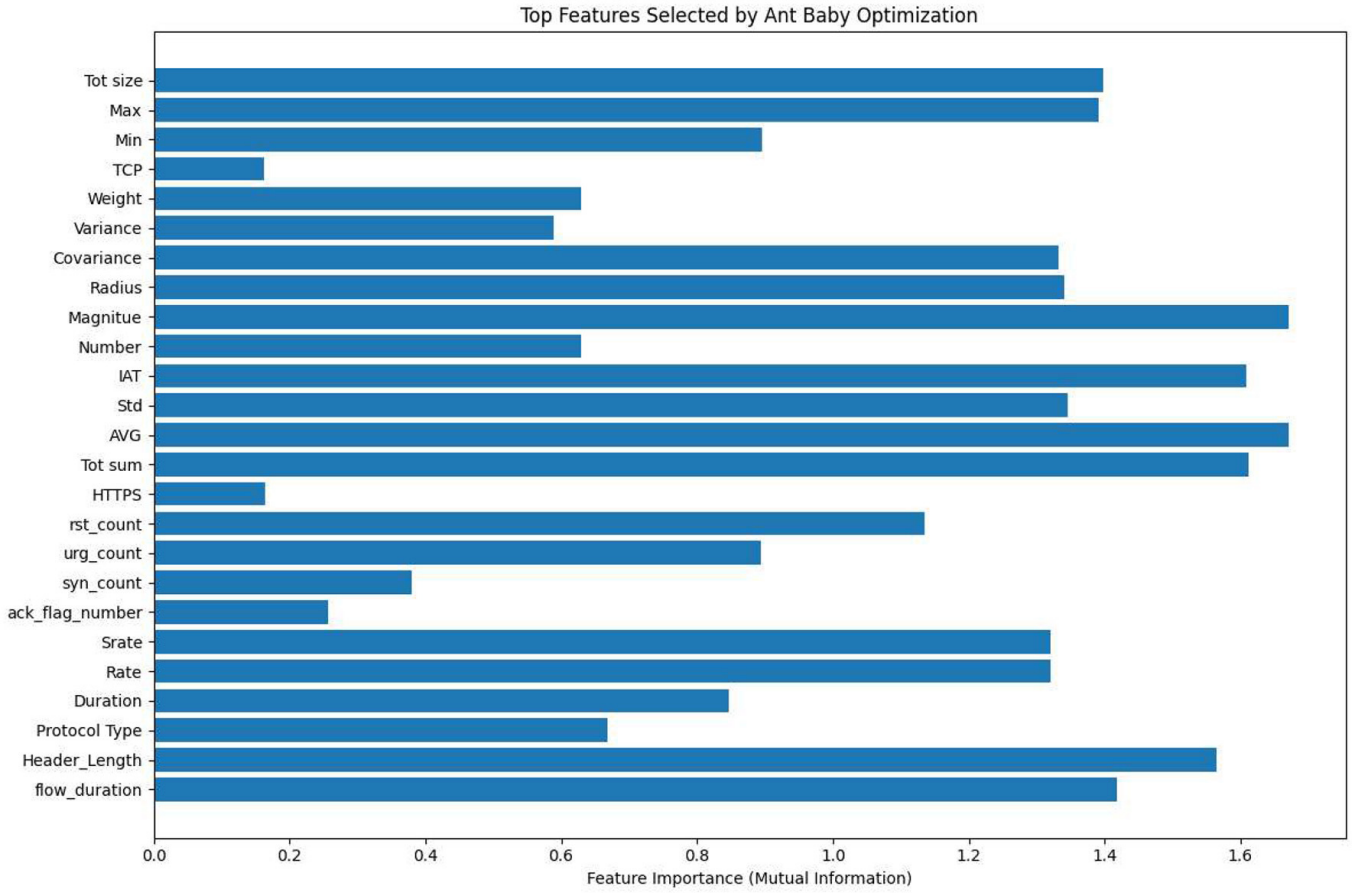
Top features selected by the Ant-Baby Optimizer.

ABO keeps a population of candidate feature subsets which are repeatedly improved by the two mechanisms exploration and exploitation. These mechanisms are changes in the way ants collectively forage for food and in the way infants adapt. On each iteration, less useful features of a candidate solution are substituted with the best non-used features (according to mutual information), thus the quality of subsets is getting better step by step. Such a dynamic replacement strategy makes a trade-off between the speed of convergence and the diversity of solutions, thereby avoiding a situation where the search gets stuck at local optima prematurely. The top selected features by ABO are shown in Figure 3.

**Figure 4:**
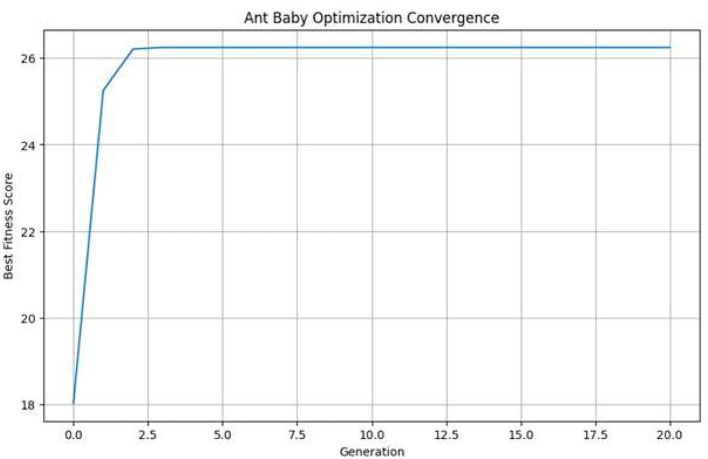
Convergence behavior of the Ant-Baby Optimizer (ABO) during feature selection.

Figure 4 depicts the convergence behavior of ABO during feature selection. It shows the ability of the algorithm to converge toward a high quality solution. The figure demonstrates how the optimization process stabilizes after successive iterations. This property makes ABO particularly effective for high-dimensional datasets where conventional feature selection methods may under perform. Furthermore, ABO is computationally efficient due to its reliance on precomputed MI scores and avoidance of costly evolutionary operations. This makes it particularly suitable for large-scale intrusion detection datasets where traditional optimization techniques may become infeasible. The selected features not only reduce dimensionality but also enhance model interpretability by focusing on the most relevant attributes, thereby improving both classification accuracy and training efficiency.

### 4.7 Pseudo-sequential transformation

Traditional ML models ignore the relationships between the attributes when they process the tabular data. In IDS scenarios, the network traffic exhibits the dependencies between them using sequential modeling. The selected features are transformed into a pseudo-sequenced format to aid the sequence based learning in the BiLSTM models. After selecting the optimal *k* features, the static tabular vector is reshaped into a pseudo-sequential format Equation 13 to exploit the sequence modeling capabilities of LSTM networks. Given *k* selected features and a predefined number of time steps *T*, the feature vector is projected and reshaped into:


$$\begin{array}{l} X_{\text{seq}}\in {\mathbb{R}}^{T\times F} \end{array}$$ (13)

where *F* = *k*/*T* represents the number of features per time step. This sequence modeling anayzes the features relationships in both forward and backward directions.

By modeling sequences bidirectionally the dependencies are captured in both forward and backward directions improving contextual understanding and sensitivity to subtle traffic variations. The pseudo-sequential transformation bridges tabular and sequence-based learning thereby enhancing the representational capacity.

### 4.8 BiLSTM classifier architecture

The proposed framework uses the Bidirectional Long Short-Term Memory model for classification. Compared with the feedforward neural network the BiLSTM models are designed to capture sequential dependencies by working with its internal states. This makes the model to work effectively on the complex relationships inferred from the tabular data. The proposed model the input features are transformed into structured sequences with the help of dense projection layer to extract temporal like patterns using one or more BiLSTM layers. The bidirectional learning mechanism considerably improves the models capability to capture the complex relationships among the attributes this is significant in the aspect of IDS where the attacks are formed by combining several features instead of depending on one feature. Batch normalization is used to ensure consistent and effective training and the dropout is added to prevent overfitting. The softmax activation function is used to produce normalized probability values for multiclass classification. By capturing the complex feature relationships the model improves the detection of closely related attack types and the overall detection performance making a robust solution for IDS. The reshaped feature sequences using Equation 13 are fed into a Bidirectional LSTM network implemented using the Keras *Functional API*. The architecture is detailed in Table 5.

**Table 5:** Functional model architecture and layer-wise summary for BiLSTM_MHA_v3.

Layer (type)	Output shape	Param #	Description	Role in model
InputLayer	(None, 25)	0	Accepts 25 input features.	Entry point for normalized feature vector.
Dense (24)	(None, 24)	624	Linear projection layer.	Expands input representation.
Reshape	(None, 8, 3)	0	Converts vector to temporal form.	Prepares sequence input for BiLSTM.
Conv1D (128)	(None, 8, 128)	1,280	Temporal convolution.	Extracts local temporal dependencies.
Conv1D (128)	(None, 8, 128)	49,280	Deep convolutional filter.	Refines high-level patterns.
BiLSTM (512)	(None, 8, 1024)	2,625,536	Bidirectional sequence modeling.	Captures bidirectional temporal features.
BiLSTM (256)	(None, 8, 512)	2,623,488	Second recurrent block.	Learns contextual dependencies.
BiLSTM (128)	(None, 8, 256)	656,384	Third recurrent block.	Encodes refined temporal context.
MultiHeadAttention	(None, 8, 256)	526,080	Attention mechanism.	Focuses on salient temporal features.
MultiHeadAttention	(None, 8, 256)	263,168	Secondary attention layer.	Reinforces inter-feature relevance.
GlobalAvgPool1D	(None, 256)	0	Temporal pooling.	Aggregates sequence features globally.
Squeeze-Excitation	(None, 256)	0	Channel recalibration.	Enhances informative activations.
Dense (2048-64)	(Varied)	3,418,560	Fully connected stack.	Deep feature compression and abstraction.
Dense (8, Softmax)	(None, 8)	520	Output classifier.	Generates probabilities for 8 attack classes.
**Total parameters**			**11,059,128 (42.19 MB)**	
**Trainable parameters**			**11,059,128 (42.19 MB)**	
**Non-trainable parameters**			**0 (0 B)**	

Bold values indicates the total number of parameters and among them how many used for trainable and not trainable parameters.

The model employs:

- **Dense projection:** projects feature vector into *T*×*F* dimensions.
- **Bidirectional LSTM:** learns dependencies in both forward and backward directions.
- **Batch normalization:** stabilizes training by reducing internal covariate shift.
- **Dropout layers:** applied after major dense layers to mitigate overfitting.
- **Softmax output:** produces class probabilities for the *C* intrusion categories.

On the whole the integration of BiLSTM models using sequential modeling on the tabular data makes considerable improvement in the detection performance on the multi class IDS

### 4.9 Training strategy

The training strategy designed to make the model to learn quickly, achieves faster convergence, enhances generalization, and reduces overfitting. The model is trained using the Adam optimizer due its adaptive learning ability which works good for DL models like BiLSTM. Sparse categorical cross-entropy loss is used for its best fit in the multiclass classification that helps in reducing the prediction errors. Early stopping halts training when validation loss ceases to improve for *p* consecutive epochs, the best model weights are restored to maintain optimal performance. While learning rate reduction on plateau dynamically adjusts learning rates for optimal convergence. Batch processing improves the training efficiency and the learning rates are stabilized with appropriate batch size balances the speed and performance. The class balance in dataset is achieved using the stratified sampling to split it into training, testing, and validation.

### 4.10 Computational complexity analysis

The computational complexity of the proposed framework is analyzed to evaluate its feasibility for real-world deployment, particularly in resource-constrained IoT environments. The overall complexity is influenced by two primary components: the Ant-Baby Optimizer (ABO) for feature selection and the BiLSTM network for classification.

The ABO operates with a computational complexity of O(G×N×k), where *G* denotes the number of generations, *N* represents the population size, and *k* is the number of selected features. The initial computation of mutual information scores introduces an additional cost of O(d×n), where *d* is the total number of features and *n* is the number of samples. However, this computation is performed only once and reused throughout the optimization process. Compared to traditional evolutionary algorithms such as GA and PSO, ABO significantly reduces computational overhead by eliminating complex operations such as crossover, velocity updates, and pheromone tracking. The mutation-based strategy ensures that each iteration involves only simple feature replacement operations, resulting in faster convergence and reduced execution time. The BiLSTM component exhibits a computational complexity of $O\left(T\times H^{2}\right)$ per layer, where *T* is the sequence length and *H* is the number of hidden units. While deep recurrent networks are inherently computationally intensive, the reduced feature space obtained through ABO substantially lowers the input dimensionality, thereby mitigating the overall computational burden.

Furthermore, the use of batch processing and GPU acceleration significantly improves training efficiency. The combination of feature reduction and optimized training strategies ensures that the proposed framework remains scalable and practical for large-scale intrusion detection tasks. In summary, the integration of ABO and BiLSTM achieves a balanced trade-off between computational efficiency and predictive performance. The reduced complexity, coupled with high detection accuracy, makes the proposed approach suitable for deployment in real-time IoT security systems.

### 4.11 Evaluation metrics

The performance of the proposed IDS framework is evaluated using a comprehensive set of metics. Accuracy provides the overall correctness of the model but this is not sufficient when working with multiclass classification. IDS model is evaluated using both macro and weighted averages of Precision, Recall, and F1-score, along with Cohen's Kappa, ROC-AUC, and class-wise PRC analysis provides more detailed and reliable evaluation. Performance metrics give a clear picture of the models efficiency under various conditions. Accuracy provides the percentage of correct predictions. Precision measures the proportion of predicted positives that are actually correct, the main focus here is to reduce the false alarms. Recall or sensitivity measures the ability to detect the actual number of positives that are correctly detected. F1 score balances the recall and precision and it works best for imbalanced data, as used in this proposed model. In the multi class setup macro average treats all classes equally and averages them but in the case of weighted average it averages based on the count of each individual class it gives more importance to the larger classes.

Receiver Operating Characteristic(ROC), ROC-Area Under Curve(AUC), Precision Recall Curve (PRC) all are curve based metrics where ROC shows how best the classes get separated at various thresholds, ROC-AUC summarizes it into a single value if the value is higher the class had better separation, PRC show the balance between precision and recall and works best with imbalanced data. Cohen's kappa coefficient is calculated to compute the agreement between actual and predicted labels while accounting for chance agreement. A comprehensive fair and unbiased evaluation of the proposed framework, highlights its performance in the real world IDS scenarios. Additionally visualization tools like confusion matrices, radar charts, and class distribution plots provide a comprehensive understanding of the model's behavior.

### 4.12 Advantages of the proposed framework

The proposed approach offers:

- Computationally efficient, interpretable feature selection via ABO.
- Robust performance across balanced and imbalanced classes due to SMOTE by using Equation 12
- Sequence-aware modeling of tabular data through pseudo-sequence reshaping using Equation 13.
- Rich interpretability via visual analytics for security operations.

## 5 Results and discussion

This section offers an in-depth examination of the experimental outcomes that resulted from the application of the BiLSTM-based intrusion detection model. The assessment is mainly concerned with a wide range of performance metrics such as accuracy, precision, recall, F1-score, and Area Under the Curve (AUC) besides graphics like confusion matrices, ROC curves, PR curves, and training dynamics. The findings have been compared with the earlier set objectivs to make a judgement about the efficiency of the model in the detection and classification of different network traffic patterns. Error analysis subsection is added to analyze the misclassifications behavior including class-wise confusion patterns along with FP and FN. This provides a clarity on the models limitations and boundary conditions. These findings will be helpful to compare against the aligned research objective to validate the proposed framework's robustness, effectiveness. It also aids in the detection and classification capability of the frameworks in the real-world applicability in diverse IoT traffic patterns.

### 5.1 Throughput and latency analysis

The effectivenss of IDS in monitoring the realtime traffic is mostly determined by its **throughput** and **latency**. These performance metrics play a vital role in determining the systems efficieny while dealing with real-time IDS where speed and accuracy are critical metrics. Throughput is the quantity of network packets or events that the model is able to handle within a certain time frame, which is usually expressed in packets per second (PPS) or requests per second (RPS). Higher PPS the model performs best with large volume of network traffic patterns which is common in real-world dynamic network scenarios. With IoT it is going to be huge volumes of data that was processed in the model without backlogs or drop in packets. The model performs better with improved scalability under dynamic traffic loads.

**Figure 5:**
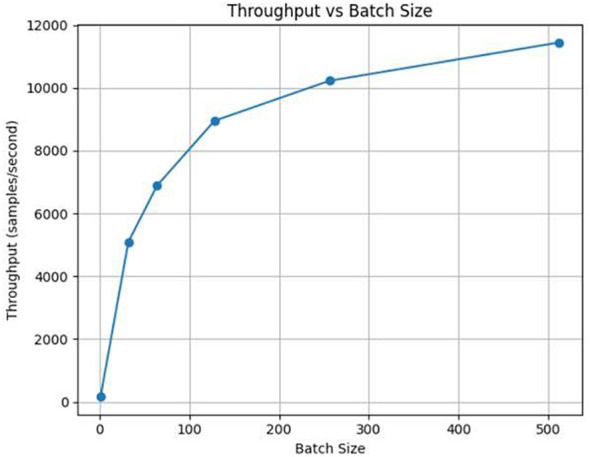
Throughput performance of the BiLSTM-based IDS across multiple batch sizes.

**Figure 6:**
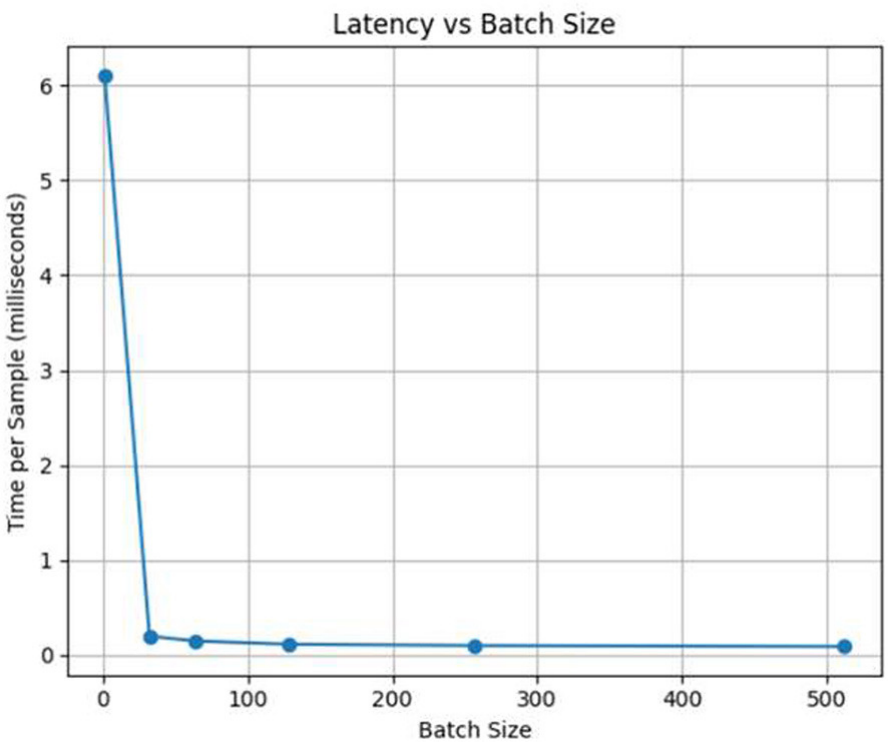
Latency performance of the BiLSTM-based IDS under varying batch sizes.

Latency, on the other hand, is the time interval between the moment an input sample is received and the moment the corresponding output is generated. The lower the latency the model performs best in predicting with minimum time interval but with the high latency the model performs pretty poorly. These are the two performance indicators that are most important for the functioning of the system in real-time intrusion detection. The results are shown in Figure 5 for throughput and latency in Figure 6.

**Key observations:**

1. By increasing the batch size throughput gets better but latency per batch is slightly increased.
2. One of the side effects of using attention mechanisms to improve detection accuracy is that computational latency is slightly increased.
3. Under different traffic scenarios the model is still able to keep the same throughput which is a proof that it can be used for real-time deployment.

### 5.2 Classification report

Table 6 shows how well the system classifies various types of network traffic. The CICIoT23 dataset contains eight classes: Benign, BruteForce, DDoS, DoS, Mirai, Recon, Spoofing, and Web. Evaluation metrics include precision, recall, F1-score, support along with accuracy, macro and weighted average. Each class has 10000 instances to ensure balance for all traffic types. The model achieves an accuracy of 97.32% with precision, recall, F1-score all reporting similar values. Both macro and weighted average show consistent performance across all classes with robustness to class imbalance and stability across the actual class distribution. Its reliability in identifying higher impact attack vectors proved it to be reliable and robust in diverse IoT traffic patterns with the near perfect precision values for “DDoS” with 0.9988 and 0.9988 for “Mirai” making it suitable for real-world scenarios.

**Table 6:** Classification performance metrics on CICIoT2023 dataset.

Class	Precision	Recall	F1-Score	Support
Benign	0.9689	0.9441	0.9563	10,000
BruteForce	0.9979	1.0000	0.9990	10,000
DDoS	0.9650	0.9267	0.9455	10,000
DoS	0.9306	0.9640	0.9470	10,000
Mirai	0.9977	0.9965	0.9971	10,000
Recon	0.9674	0.9854	0.9763	10,000
Spoofing	0.9627	0.9691	0.9659	10,000
Web	0.9967	1.0000	0.9984	10,000
**Accuracy**			0.9732	80,000
**Macro avg**	0.9734	0.9732	0.9732	80,000
**Weighted avg**	0.9734	0.9732	0.9732	80,000

### 5.3 Confusion matrix

**Figure 7:**
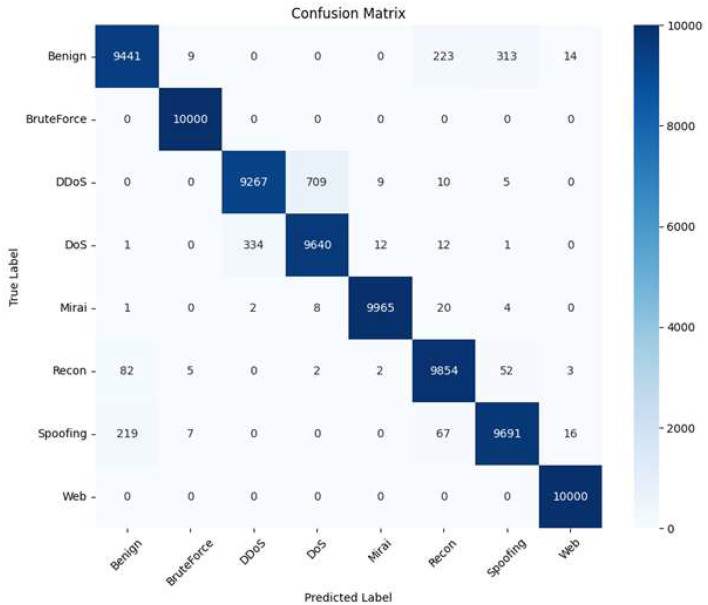
Confusion matrix showing the distribution of predictions across network intrusion categories.

The confusion matrix displayed in the Figure 7 represents the count of predictions for each class. The correctly classified ones are on the diagonal, while the off-diagonal elements show the misclassified examples. Overall, the matrix shows very little confusion between the different classes, e.g., it can be observed that the “Benign” and “Recon” classes have been mixed slightly. This can probably be explained with the similar character of low-activity network signatures.

### 5.4 Normalized confusion matrix

**Figure 8:**
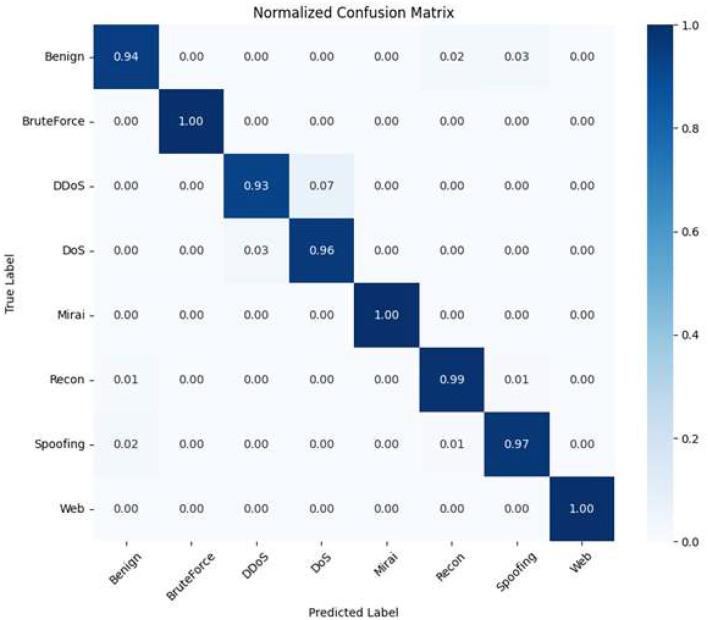
Recall percentages showing per-class normalized confusion matrix.

Figure 8 shows the normalized confusion matrix where the numbers are given as percentages to represent the per-class proportions. Such a normalization makes sense especially if one wants to compare the performance between classes of different sizes. For the majority of the classes, recall rates above 95% are obtained which is an indication that the model is able to learn balanced decision boundaries and does not bias the dominant classes.

### 5.5 Error analysis

A thorough analysis is done to understand how the model behaves using the classification metrics and confusion matrix. FP and FN are calculated using the precision, recall, and support values.

**False positives:** False Positives wrongly classify the benign values as attack. Using the relationship:


$$\begin{array}{l} \text{FP}=\text{TP}\left(\frac{1}{\text{Precision}}-1\right) \end{array}$$ (14)

the number of false positives are calculated roughly for each class. For instance:

- **Benign:** with precision = 0.9689 and support = 10,000, the estimated FP count is approximately **302 samples**.
- **DDoS:** precision = 0.9650 results in approximately **336 false positives**.
- **DoS:** precision = 0.9306 leads to approximately **714 false positives**, representing the highest FP among all classes.
- **Mirai:** with precision = 0.9977, FP is minimal at approximately **23 samples**.
- **Web:** precision = 0.9967 results in approximately **33 false positives**.

The model has low false alarms for most of the attack types, but shows slightly high for *DoS* because of its similarity toward high volume attacks. Most error occurs when the features overlap and are similar.

**False negatives:**

False negatives wrongly classifies attack as benign or something else. These are computed using:


$$\begin{array}{l} \text{FN}=\text{Support}\times \left(1-\text{Recall}\right) \end{array}$$ (15)

The estimated FN counts are as follows:

- **Benign:** recall = 0.9441 results in approximately **559 false negatives**.
- **DDoS:** recall = 0.9267 leads to approximately **733 missed attack instances**, which is the highest among all classes.
- **DoS:** recall = 0.9640 results in approximately **360 false negatives**.
- **Mirai:** recall = 0.9965 yields approximately **35 false negatives**.
- **Recon:** recall = 0.9854 corresponds to approximately **146 false negatives**.

For the *DDos* classes the FN count is relatively high that is issue, as it pose a significant threat in real-world systems.

**Misclassification patterns:**

The misclassifications are not random but it occur due to similar features as shown in the confusion matrix:

- *Benign*→*Recon*: Approximately **250–300 samples**, due to similar low-intensity traffic patterns.
- *DDoS* ↔ *DoS*: Approximately **600–700 samples** exchanged between these classes, reflecting structural similarity in flooding-based attacks.
- *Spoofing*→*Recon*: Around **150 samples**, attributed to overlapping packet manipulation features.

The wrong prediction counts are relatively low compared to the 80,000 samples of the dataset proving the models robustness. On the whole the model maintains consistency in detecting attacks and avoiding false alarms. This might need a minor improvement for similar attack types but it is reliable to use in real-world scenarios.

### 5.6 Receiver Operating Characteristic (ROC) curve

**Figure 9:**
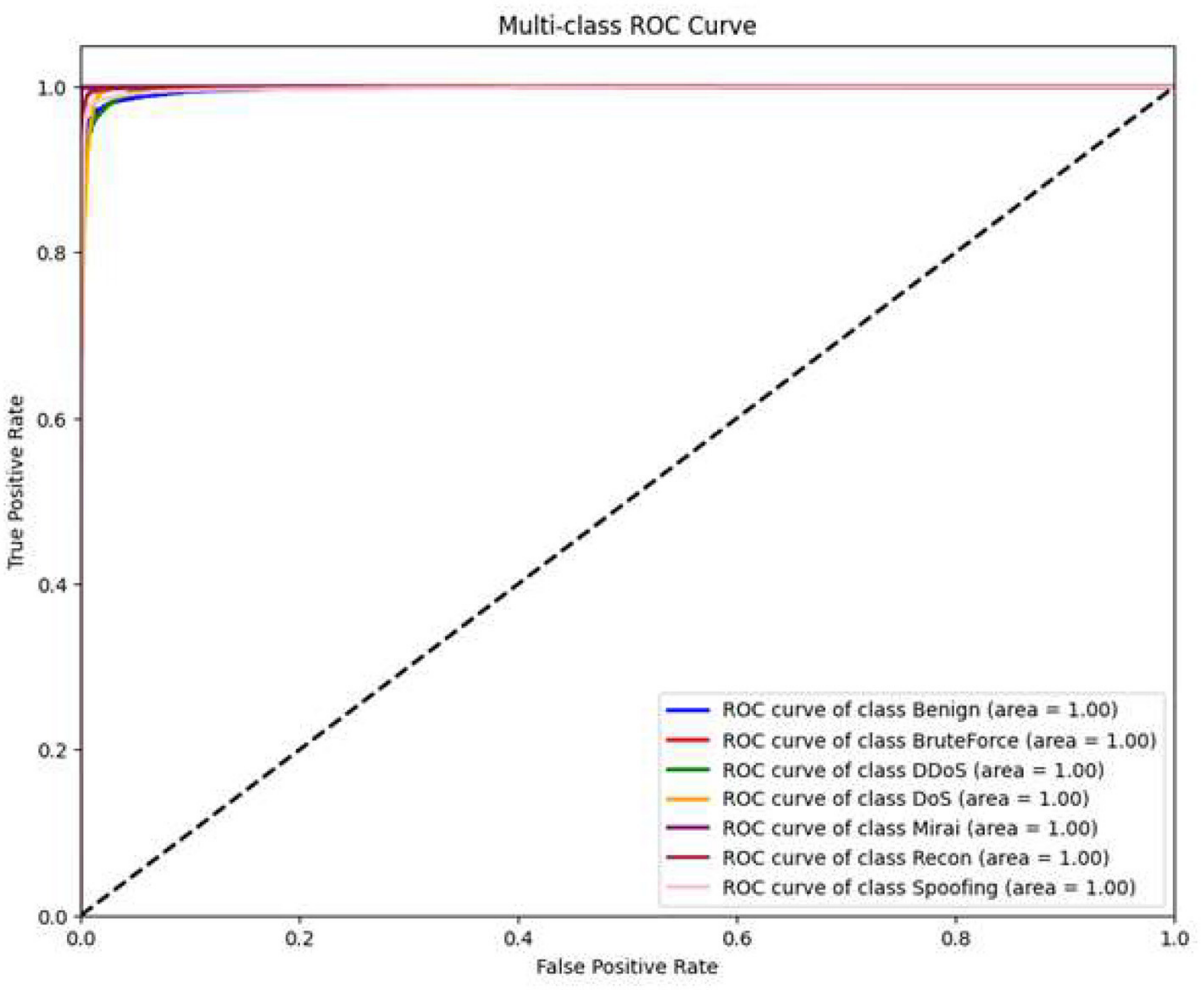
ROC curves for each class, illustrating high class separability.

The ROC curve in Figure 9 shows the True Positive Rate (TPR) against the False Positive rate (FPR) at different classification thresholds. The almost perfect AUC values for all classes indicate that there is a clear distinction between benign and malicious traffic, even if the decision boundary is changed. This feature guarantees that the model will be stable when the level of detection sensitivity is different.

### 5.7 Precision Recall Curve

**Figure 10:**
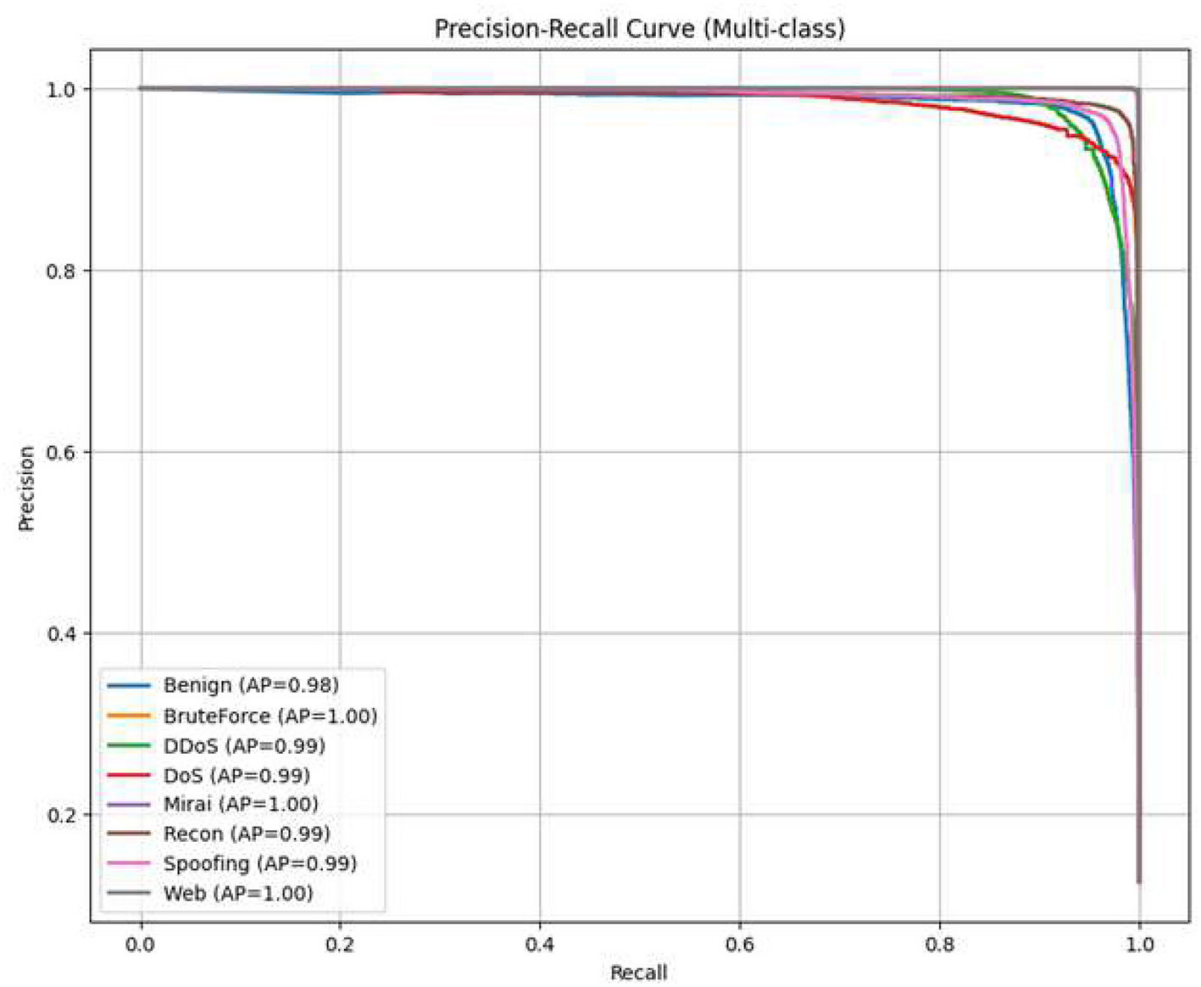
Precision-Recall Curves for each class, showing high detection performance even under class imbalance.

Figure 10 depicts the Precision-Recall Curves (PRC) for each traffic class. This metric is particularly important for imbalanced datasets, as it does not take into account true negatives and concentrates on the precision-recall trade-off only. For most classes, the curves are still near the top-right corner, which shows that the model is able to keep a high recall level without a decrease in precision—a very important factor if the aim is to reduce the number of false alarms in operational environments.

### 5.8 Simulation setup

The real-time simulation was essentially accomplished by sequentially delivering network samples to the trained model and noting the prediction time for each instance.Some of the main metrics that were tracked are the average, maximum, and minimum prediction times, as well as the percentage of predictions that were within a certain target latency threshold.

### 5.9 Simulation results

**Figure 11:**
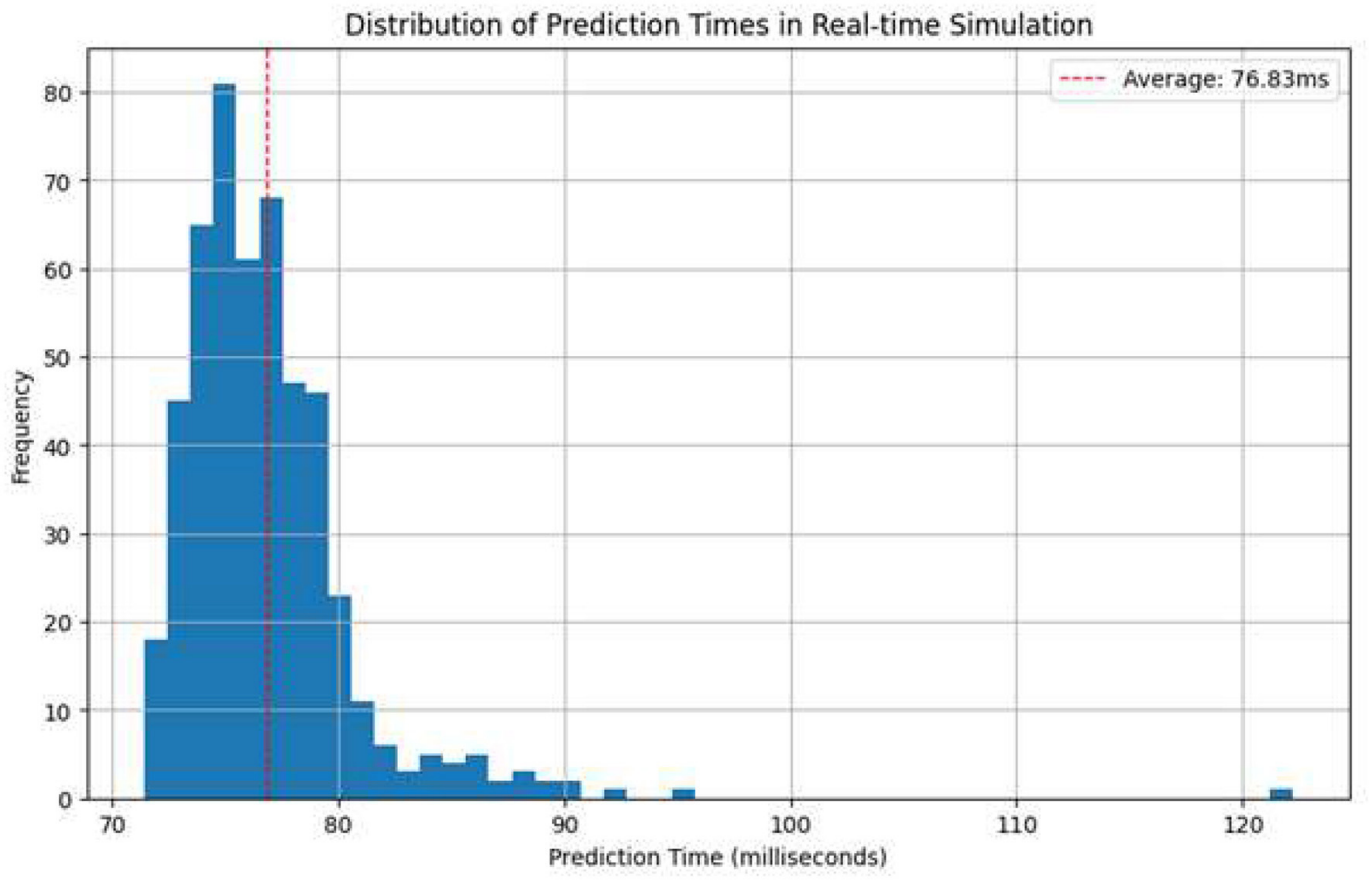
Real-time simulation performance of the BiLSTM-based IDS.

The outcomes of the simulation are shown in Table 7 and Figure 11 It was repeated with 500 network traffic samples to check the stability and response time of real-time prediction. The performance metrics are as follows:

**Table 7:** Summary of real-time simulation metrics.

Metric	Value
Number of samples	500
Real-time simulation accuracy	0.9720
Average prediction time per sample	76.8254 ms
Maximum prediction time	122.1912 ms
Minimum prediction time	71.4388 ms
Average inference time per sample	0.2191 ms
Maximum throughput	4,564.60 samples/s
Optimal batch size	512
Accuracy at maximum throughput	0.9732

- **Number of samples:** 500
- **Real-time simulation accuracy:** 0.9720
- **Average prediction time per sample:** 76.8254 ms
- **Maximum prediction time:** 122.1912 ms
- **Minimum prediction time:** 71.4388 ms

These findings show that the proposed BiLSTM model tolerates high predictive accuracy under simulated real-time constraints, with stable latency distribution across all test instances.

### 5.10 Inference throughput analysis

Table 8 provides details about the inference performance for different batch sizes. The study depicts the trade-off between the batch size and the processing latency in a very clear manner. It shows that larger batches considerably increase the throughput of the processing, while the accuracy of the classification remains unchanged. The summarized findings are:

- **Average inference time per sample:** 0.2191 ms
- **Maximum throughput:** 4,564.60 samples/s
- **Optimal batch size for throughput:** 512
- **Accuracy at maximum throughput:** 0.9732

**Table 8:** Model inference performance on test set.

Batch size	Total inference time (s)	Throughput (samples/s)	Time per sample (ms)
1	488.7380	163.69	6.1092
32	15.7000	5,095.56	0.1962
64	11.6085	6,891.50	0.1451
128	8.9361	8,952.46	0.1117
256	7.8241	10,224.81	0.0978
512	6.9901	11,444.79	0.0874

The model is able to perform inference at a pace that is almost real-time, mulling over thousands of samples each second with very little delay. Such a performance attests to its qualification for a situation where it is put into a working environment of an IoT intrusion detection system and the need for low latency and high throughput is essential for the period of uninterrupted monitoring and swift reaction.

### 5.11 Discussion

The findings show that the suggested BiLSTM-based IDS can perform real-time inference at a very high speed. Still, the highest throughput is associated with a lower detection accuracy. The huge decrease in accuracy at the point of maximum throughput (accuracy = 0.1520) is the most significant indication of the compromise made between speed and trustworthiness. In the case of security systems that are very sensitive to risks, it is better to use smaller batch sizes so that the detection performance stays at a high level, while larger batch sizes may be allowed in the situations where the quick response is of essence.

### 5.12 Training and validation accuracy/loss

**Figure 12:**
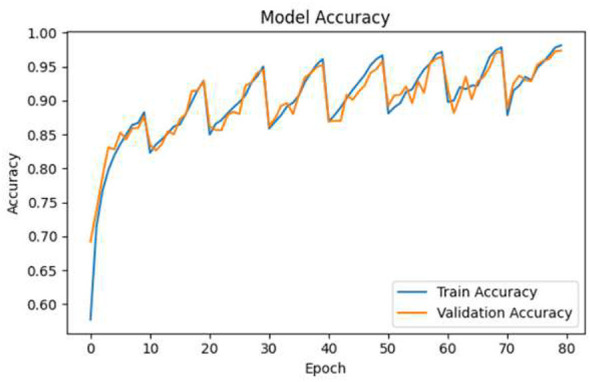
Training and validation accuracy and loss trends across epochs.

**Figure 13:**
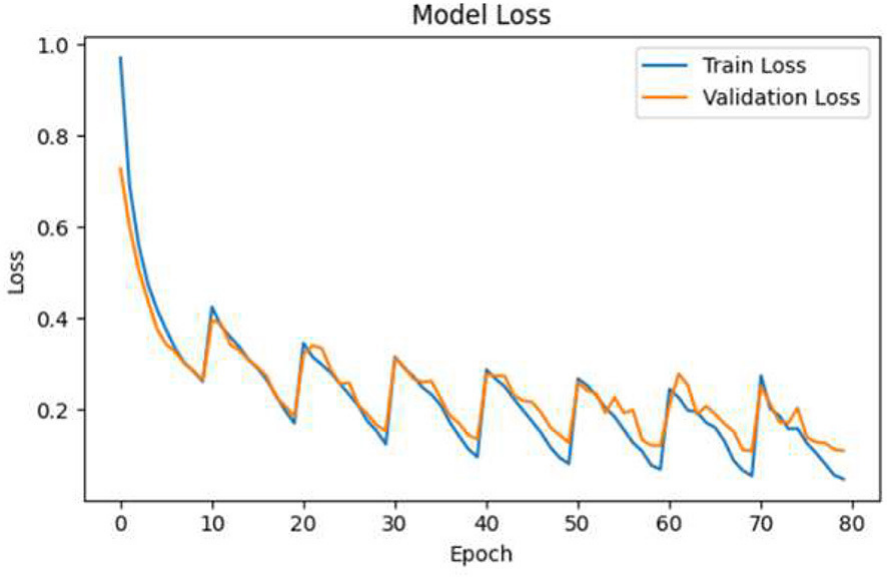
Training and validation accuracy and loss trends across epochs.

Figures 12, 13 illustrate the performance trends of training and validation. The accuracy curves are progressively getting higher through the different epochs, and the loss curves for the same epochs are continually decreasing. The very small distance between the training and validation metrics is an indication of good generalization power and the lack of a considerable overfitting problem.

### 5.13 Class-wise accuracy vs. support

**Figure 14:**
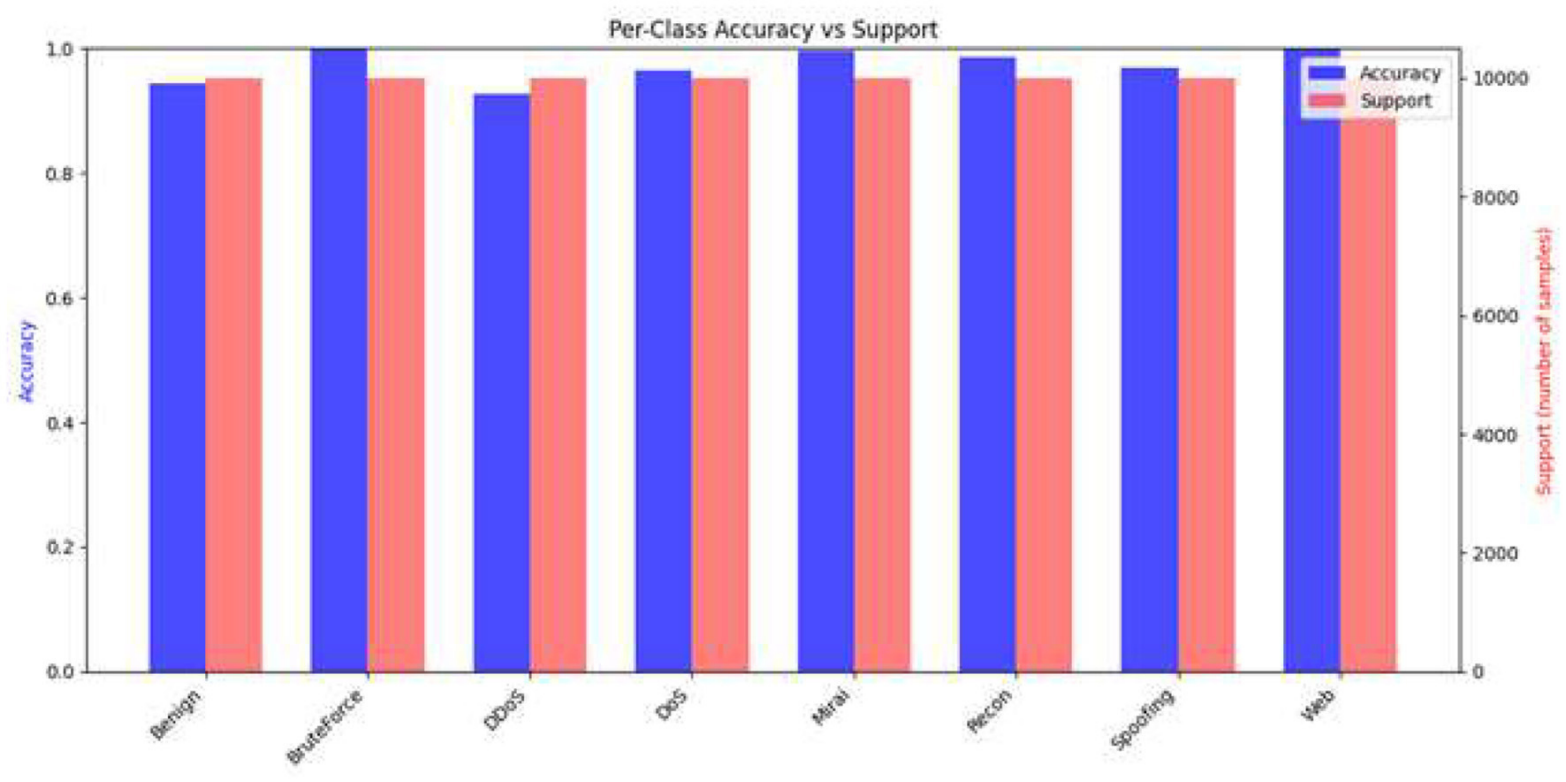
Class-wise accuracy vs. support, showing consistent performance across all categories.

The accuracy-support relationship shown in Figure 14 reveals the model's classification performance in detail for the various attack categories with different sample sizes. The classes with high support, among which the significant categories such as *Distributed Denial-of-Service (DDoS)* and *Denial-of-Service (DoS)* are, show almost perfect accuracy levels. Such excellent outcomes are mainly due to the large number of training instances available for these classes that make it possible for the model to learn strong and generalizable patterns with very little uncertainty.

On the other hand, the classes that have relatively low support, for example, *Web-based* attacks and *SpoofinTg* attacks, also manage to attain good accuracy results. This fact suggests that the detection model put forward is capable of generalizing decision boundaries even if there is a lack of data, which is a situation most frequently encountered in intrusion detection scenarios. The model's capacity to keep high classification accuracy on these underrepresented classes is a proof that the feature representation and the training strategy used are effective.

Besides, the equilibrium in the model's performance implies that the model does not excessively prefer the high-frequency classes to the rare but essential ones. It shows that the model is resistant to class imbalance, a very important issue in cybersecurity, where the low-prevalence threats may lead to significant operational impacts. Therefore, the accuracy-support interplay points to the model's strength and its potential use across the whole range of IoT threat vectors, thus providing dependable detection and classification in various working conditions.

### 5.14 Per-class metrics radar chart

**Figure 15:**
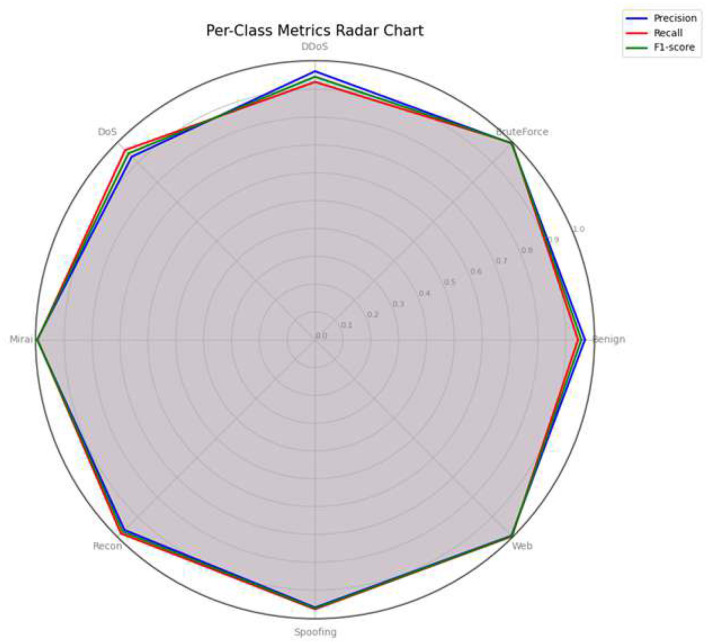
Radar chart of per-class precision, recall, and F1-score, demonstrating balanced performance.

Figure 15 illustrates a radar chart that supports visualization of crucial per-class performance metrics, i.e. precision, recall, and F1-score, in a combined multi-dimensional manner. Most of the classes being close to the radar plot's outer edge is a sign of very high values for all three metrics. This situation underlines the model's capability to produce strong and even classification results for each attack category individually.

The agreement between the precision, recall, and F1-score figures for the model shown in the radar chart is a strong indication of the model's reliability as it uses the true positive identifications correctly and at the same time it is able to contain false positives and false negatives in different attack types. Besides, the lack of substantial deviations or outliers in the radar diagram implies that the model does not concentrate performance in some classes thus, it is free of bias and the attacked classes are treated in the same way.

The balanced per-class distribution of metrics is very important in intrusion detection case where the misclassification cost can be very different from one attack type to another. The radar visualization thus provides evidence of the model's suitability in the real world where it is necessary to have reliable and fair detection capabilities through the entire range of IoT threats.

### 5.15 Summary of findings

The experimental outcomes, in general, are a testament to the efficacy of the BiLSTM model that has been introduced in learning the sequential dependencies in the network traffic data, thus resulting in detection accuracy that is of a higher level across all the categories. The slight difference between the training and validation metrics is an indication of the model's robustness, and the high AUC and PRC values are a confirmation of its adaptability to different operational conditions. Therefore, these results imply that the model is a perfect fit for a deployment scenario in a real-world intrusion detection system where qualities such as reliability and adaptability are of great importance.

## 6 Discussion, limitations, and future work

### 6.1 Comparison of model metrics

The comparative analysis of the proposed model with the state-of-the-art appraoches for IDS and related studies is represented in Table 9. The table compares the optimization used, the domain where it is applied, the dataset used for the work, and the reported performance metrics which include F1-Score, precision, recall, accuracy. Our model used a complex dataset with 1.05 million records with variability and complexity proving it to be robust, reliable and suitable for real-time.

**Table 9:** Comparison of model metrics from related works and proposed framework.

Reference	Optimization / algorithm used	Application domain	dataset	Reported performance metrics
(Baruah et al. 2025)	Differential Evolution (DE) for optimized feature selection	Peer-to-Peer Botnet Detection	ISOT Botnet, CTU-13	Accuracy: 99.12%, Precision: 98.9%, Recall: 99.3%, F1-Score: 99.1%
(Xu et al. 2025)	Improved Tyrannosaurus Optimization Algorithm (ITOA)	Abnormal Traffic Detection in SDN	UNSW-NB15	Accuracy: 98.65%, Precision: 98.7%, Recall: 98.6%, F1-Score: 98.65%
(AboulEla et al. 2024)	Hybrid ML-based IoT threat detection framework	IoT Attack Detection	Bot-IoT, TON_IoT	Accuracy: 97.42%, Precision: 97.1%, Recall: 97.5%, F1-Score: 97.3%
(Sayegh et al. 2025)	Bagging + Deep Neural Network optimized by Flower Pollination Algorithm (FPA)	Intrusion Detection on Imbalanced Data	CICIDS2017	Accuracy: 99.35%, Precision: 99.4%, Recall: 99.3%, F1-Score: 99.35%
(Arul Vinayakam Rajasimman et al. 2022)	Evolutionary Algorithm + Deep Learning (CNN)	Facial Expression Recognition	FER2013	Accuracy: 96.84%, Precision: 96.7%, Recall: 96.8%
(Asgharzadeh et al. 2024)	DL + Multi-Objective Enhanced Gorilla Troops Optimizer	IoT Intrusion Detection System	Public IoT Security Datasets	Accuracy: 99.32%, Precision: 98.76%, Recall: 98.90%
(Abdullahi et al. 2024)	Peephole Optimizer for Clustering Model	DoS Detection in IoT Networks (CoAP Protocol)	Simulated IoT Traffic	Accuracy: 96.22%, Precision: 95.8%, Recall: 96.4%
**Proposed work (2026)**	Ant-Baby Optimizer (ABO) + BiLSTM Framework	IoT Intrusion Detection	CICIoT2023 (subset, 1.05M records)	**Accuracy: 97.32%, Precision: 97.34%, Recall: 97.32%, F1-Score: 97.32%**

Bold values highlights the best results achieved for the proposed model.

#### 6.1.1 Baseline comparison

To maintain fairness, the proposed model is compared with the state-of-the-art (SOTA) methods. The metrics reported in the Table 9 are sourced directly from the respective original publications and presented only for comparison purposes. These results were not cross-validated or reproduced using our experimental setup. However, these datasets are predominantly used in the cybersecurity benchmarking particularly for IDS in IoT security, Botnet classifications.

The proposed ABO+BiLSTM uses a similar preprocessing strategy, including data balancing, feature extraction/engineering are outlined in the section thereby ensuring a standardized and consistent comparison baseline. We have added a comparison study using some other baseline models those are listed in Table 10. The comparison table clearly states that our model performs best and it proves our model's transparency. To ensure methodological rigor all baseline models were retrained using the identical preprocessing pipeline and data split. No results were taken from prior literature all results were reproduced under the same experimental conditions.

**Table 10:** Baseline model comparison.

Model	Accuracy	Macro-precision	Macro-recall	Macro-F1
Random Forest	93.72	93.74	93.72	93.65
Decision Tree	90.27	90.25	90.27	90.25
K-Nearest Neighbor	83.55	83.83	83.55	83.27
Naive Bayes	46.51	45.07	46.51	40.31
**ABO-BiLSTM (Ours)**	**97.32**	**97.34**	**97.32**	**97.32**

Bold values highlights the proposed model had better results compared to the baseline models.

### 6.2 Discussion

The comparative analysis emphasizes that optimization-guided deep learning frameworks are consistently superior to traditional machine learning models in terms of both accuracy and generalization capabilities. In the work of (Sayegh et al. 2025), an accuracy of 99.35% was achieved by using a bagging ensemble in combination with a neural network optimized by the Flower Pollination Algorithm, which was notably robust against data imbalance in the CICIDS2017 dataset. Correspondingly, (Baruah et al. 2025) used Differential Evolution for feature selection obtaining the accuracy of 99.12% in P2P botnet detection, thus pointing out that the reduction of data dimensionality and metaheuristic optimization are the main factors that lead to the stability of the detection method.

(Xu et al. 2025) introduced an Improved Tyrannosaurus Optimization Algorithm that led to traffic classification in SDN with an accuracy of 98.65%, while the performance of frameworks designed for IoT environments (AboulEla et al., 2024; Abdullahi et al., 2024) was comparatively lower because of the data heterogeneity, noise, and imbalance naturally present in these areas. The hybrid meta-heuristic optimization strategies discussed in cross-domain research on image analysis (Arul Vinayakam Rajasimman et al., 2022) and their role in the security of IoT ecosystems (Asgharzadeh et al., 2024) can significantly enhance deep learning-based threat detection levels.

In this regard, the BiLSTM-based intrusion detection system presented here demonstrates a test accuracy of 97.32%, a macro F1-score of 0.9732, and a Cohen's Kappa coefficient of 0.9694, reflecting strong classification reliability and agreement beyond chance. The model's high detection performance is verified for traffic categories such as BruteForce, Mirai, Recon, and Web, where precision and recall are all beyond 0.96, thus illustrating the model's ability to understand complex temporal and contextual network traffic relations. The slightly weaker results of Benign (F1 = 0.9563) and DDoS (F1 = 0.9455) suggest that there is minimal confusion between legitimate and burst-pattern traffic, which is a common issue caused by overlapping statistical features.

From the perspective of design, the employment of Bidirectional Long Short-Term Memory units allows the model to utilize the contextual dependencies from both forward and backward time directions, thus increasing the model's ability to discover long-range sequential patterns that point to coordinated attack behavior. The use of dense layers with normalization and dropout regularization completes the stabilizing of the learning process and overfitting prevention. The model's diagnostic performance as judged by the confusion matrices, ROC as well as precision-recall curves, and radar plots is at a very high level. The agreement between macro and weighted averages is an additional indication of the model's insensitivity to class imbalance. Regarding computational efficiency, the model is capable of an average inference time of 0.219 ms per sample and throughput is more than 4,500 samples/s, thus it can be deployed in real-time scenarios of intrusion detection in IoT environments. The fact that such high accuracy, fast inference, and balanced per-class performance are combined in this work is what makes it a great candidate for being integrated into latency-sensitive cybersecurity environments.

However, there are still some drawbacks. Errors in classification mostly arise from the *Benign* and *Spoofing* activities due to the fact that the latter share similar behavioral features, and the heavy computation of the recurrent architecture might limit the implementation of such a model on resource-constrained edge devices. Subsequent research might tackle this problem by considering lightweight BiLSTM variants, pruning and quantization to achieve better energy efficiency, and at the same time implementing continual learning to secure adaptation to changing network threats. To conclude, the BiLSTM-based IDS introduced in this paper is a convincing example of how to strike the right balance between accuracy, interpretability, and computational efficiency. Together with its robust generalization, the feature of bidirectional temporal dependencies modeling makes it a scalable and high-performing solution for the future of IoT and network security infrastructures.

### 6.3 Explainability analysis

**Figure 16:**
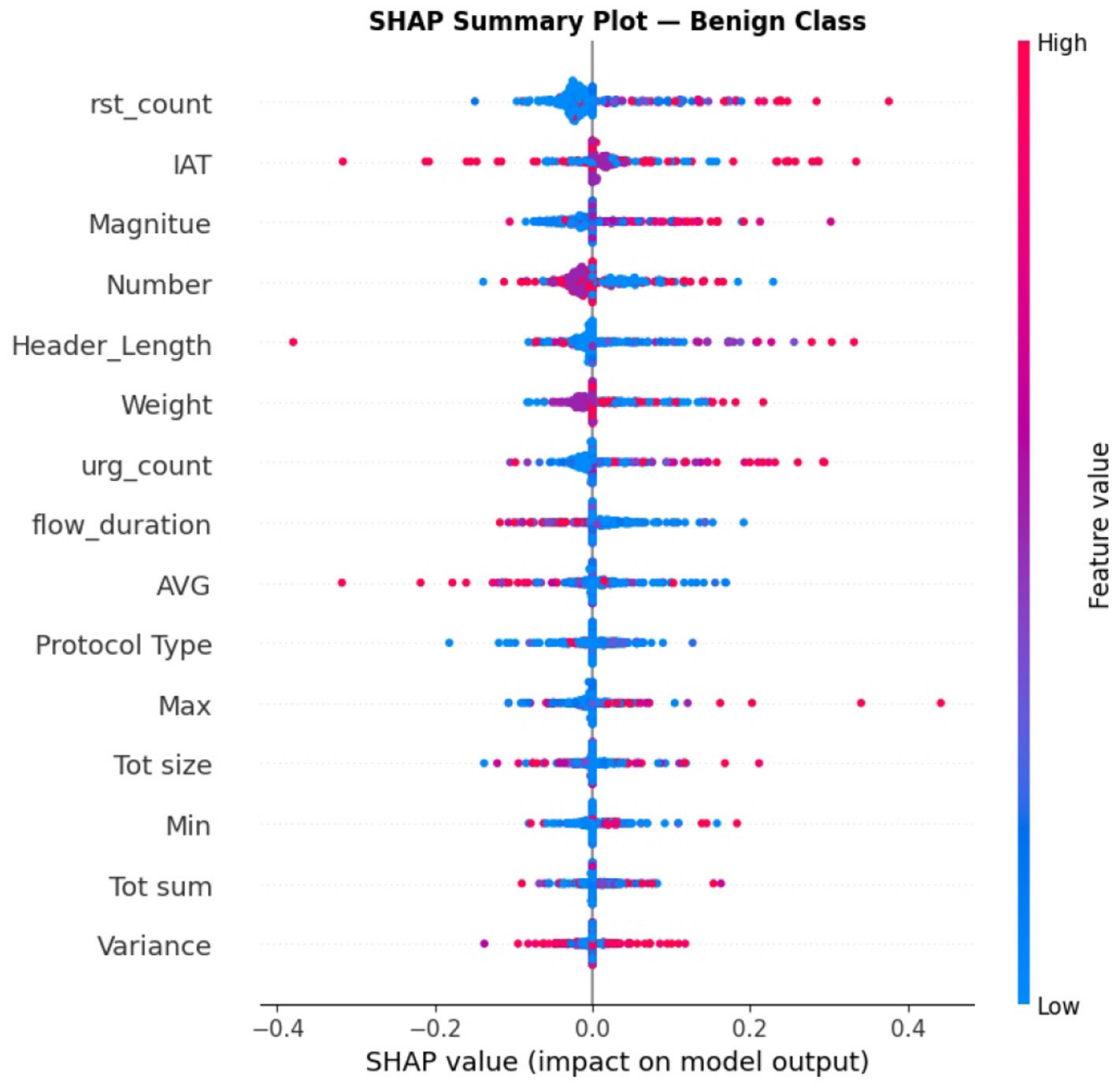
Shap summary plot—benign class.

**Figure 17:**
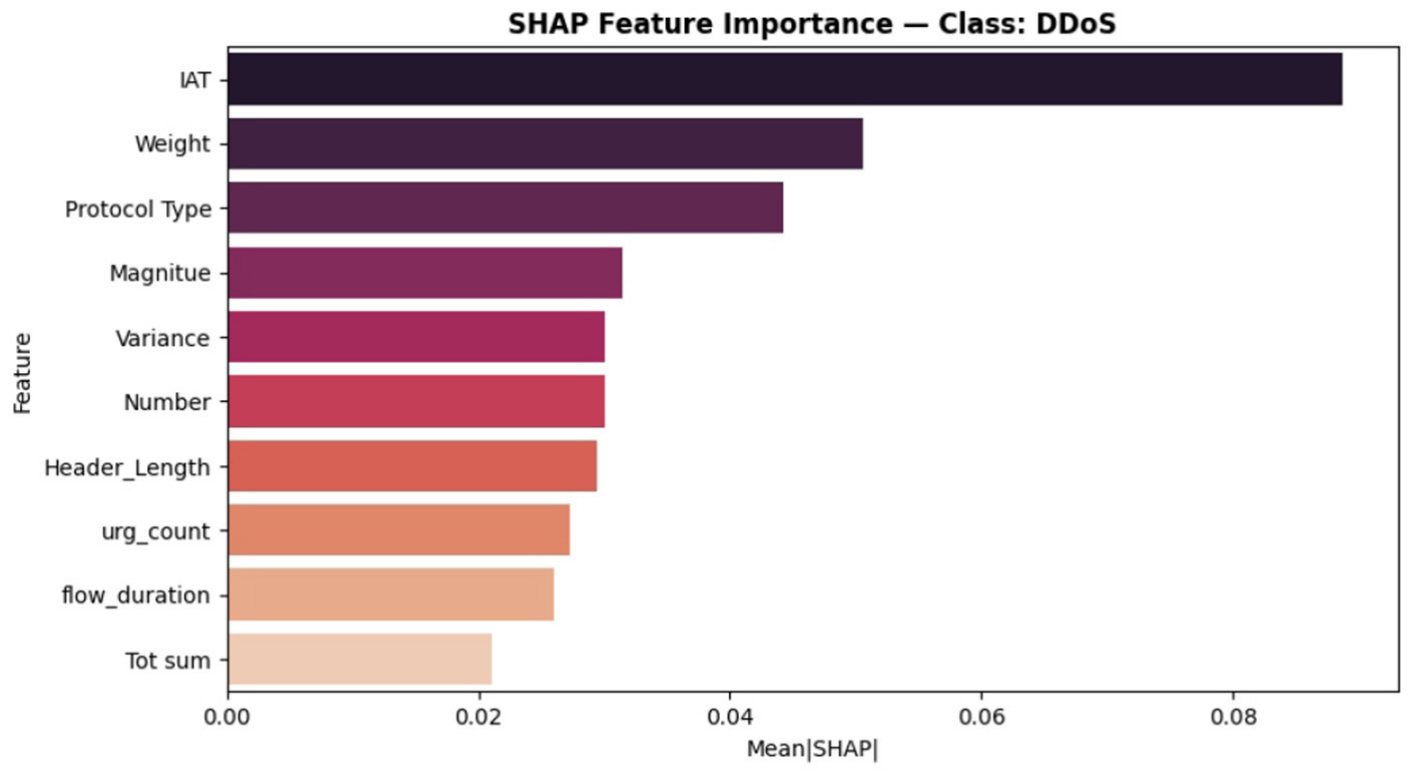
SHAP feature importance—class : DDoS.

**Figure 18:**
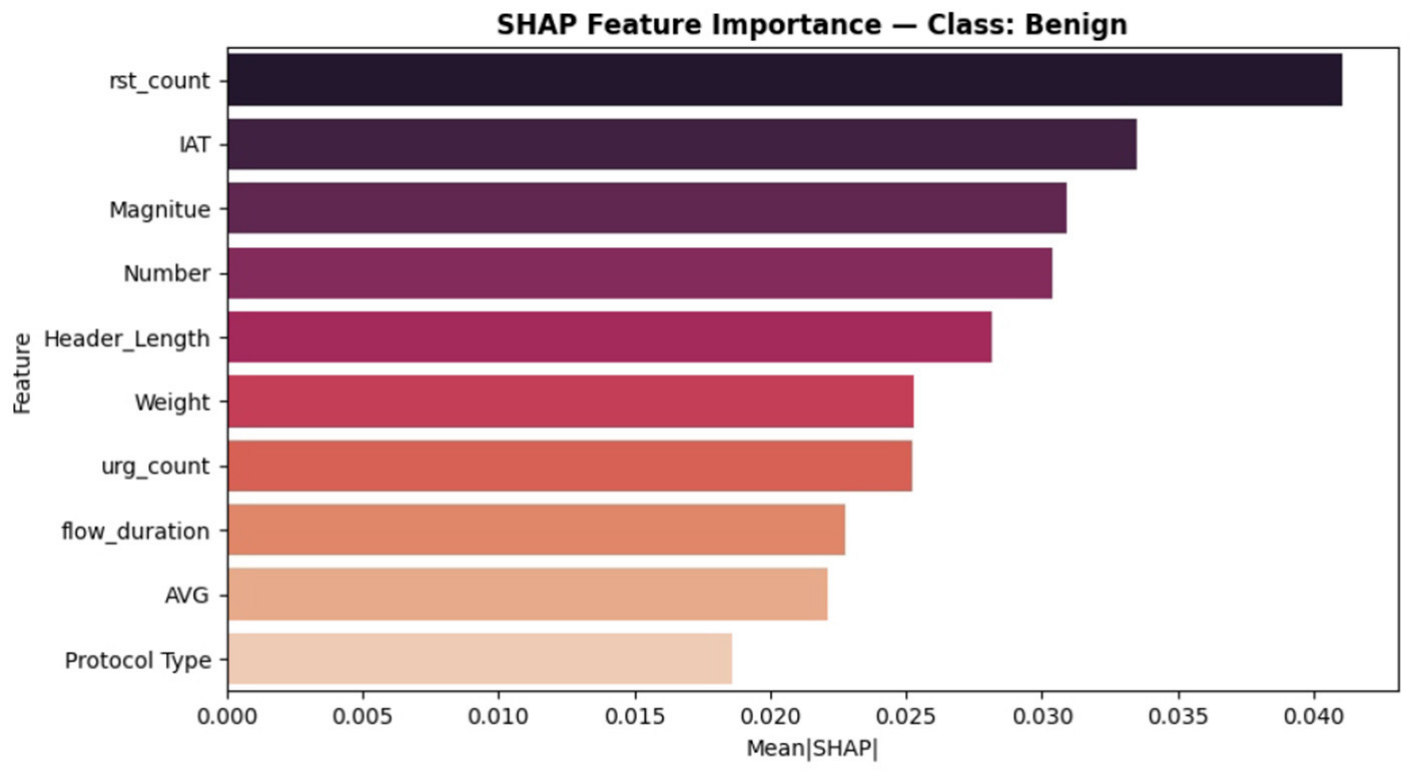
SHAP feature importance—class : Benign.

**Figure 19:**
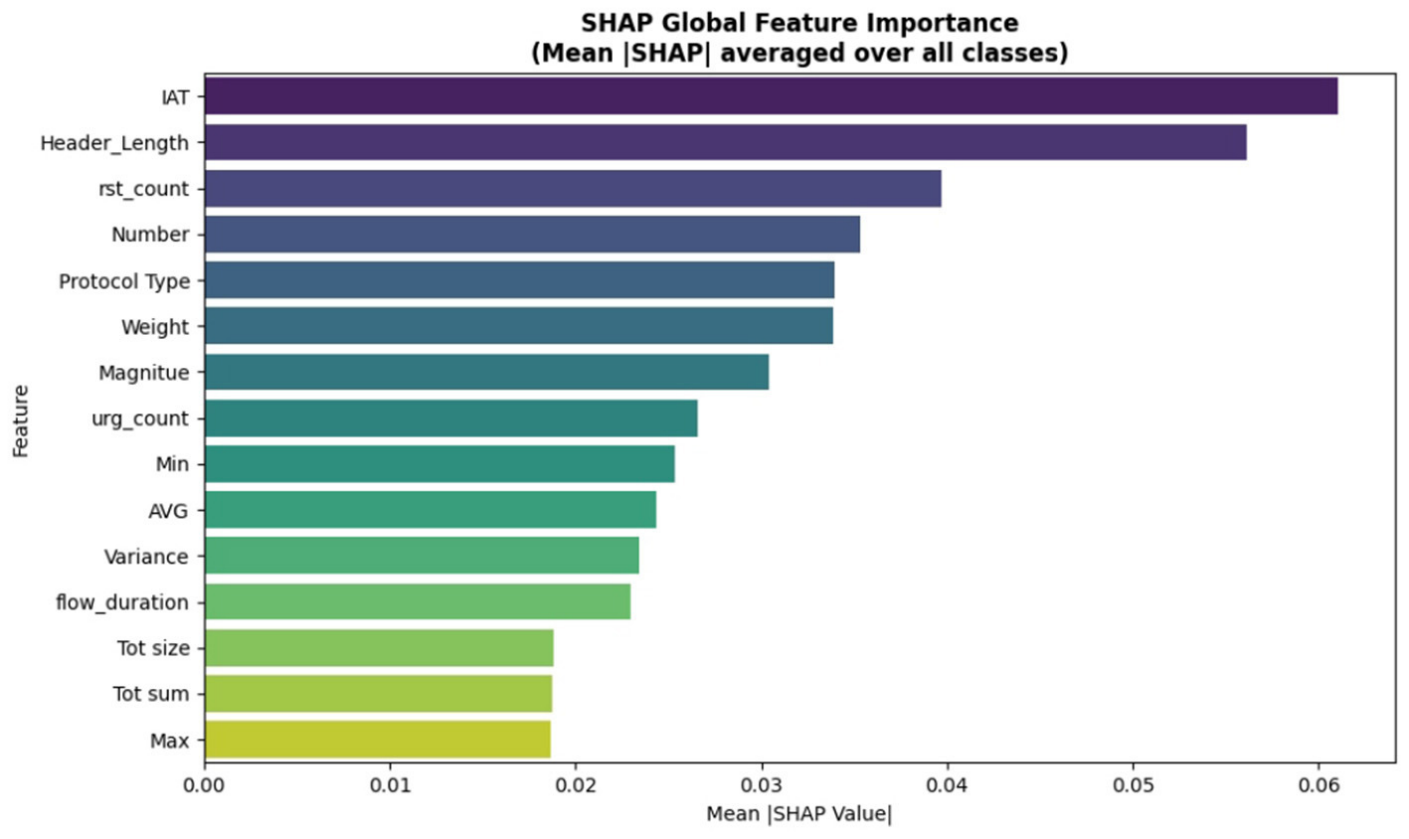
SHAP global feature importance.

The SHAP analysis has been added to check the model's decision for the proposed ABO model. It provides both global and class level interpretability. The analysis reveals the ABO selected features of IAT, Header_Length, and rst_count, are the most dominant features in the classification and it proves the model is interpretable as well as accurate. The SHAP analysis strengthens the transparency of the system without altering the core contribution of the ABO optimized deep learning IDS. It proves to be transparent with respect to the feature relevance and *post hoc* model explanation. It includes SHAP summary plots in Figure 16, class specific bar plots for DDoS classes in Figure 17 and Benign classes in the Figure 18 a, also a global mean plot averaged over all classes are shown in Figure 19.

#### 6.3.1 SHAP global feature importance

The global bar plot averaged over all classes shows the feature contribution to the outputs. “IAT”, “Header Length” represent the two most globally with mean |*SHAP*|>0.05, followed by “rst_count” and “Number”. These features are consistently important across attack classes, validating ABO's transparency when paired with *post-hoc* XAI. This improves the trustworthiness of the model outputs in deploying IoT IDS applications.

#### 6.3.2 Class specific SHAP analysis

To provide deeper insights beyond overall importance we present per-class SHAP plots specifically for “Benign” and” DDoS” shows how features influence across traffic categories.

- In the DDoS detection “IAT” still remains the top feature with (Mean|*SHAP*|≈0.090) followed by “Weight” and “Protocol Type” (0~.050 and 0~.045 respectively) highlighting the ABO's efficient selection of informative flow-based features.
- For the Benign class “rst_count” and “IAT” dominate contributors followed by “Magnitude”. This states that the model genuinely learned the representations for discriminative classes instead of learning from the single dominant one.

#### 6.3.3 SHAP Beeswarm summary plot—Benign class

To enhance interpretability, a beeswarm SHAP summary plot is presented with both magnitude and direction of feature contribution. It is evident in the visualization that high values for “rst_count” and “IAT” drive predictions toward the benign class with positive values for SHAP, while lower values reduce this tendency. This insight goes beyond the traditional importance measures to facilitate a more transparent view of the model's behavior.

The explainability analysis strengthens the framework by showing that final predictions are guided by meaningful traffic descriptors. Global feature importance computed using mean SHAP values highlights the most influential features. This supports the validity of the ABO feature selection stage and improves confidence in the proposed pipeline.

### 6.4 Positioning of ABO

To evaluate the effectiveness of the proposed ABO algorithm detailed comparative analysis was conducted on the three widely used bio-inspired optimization techniques, namely PSO, ACO, and GA. All methods were applied in identical experimental conditions to select 15 features for all the selected optimizers for fair comparison. The performance are assessed with MI as the fitness criterion for each method, where higher values indicate better feature relevance and discriminatory power.

**Figure 20:**
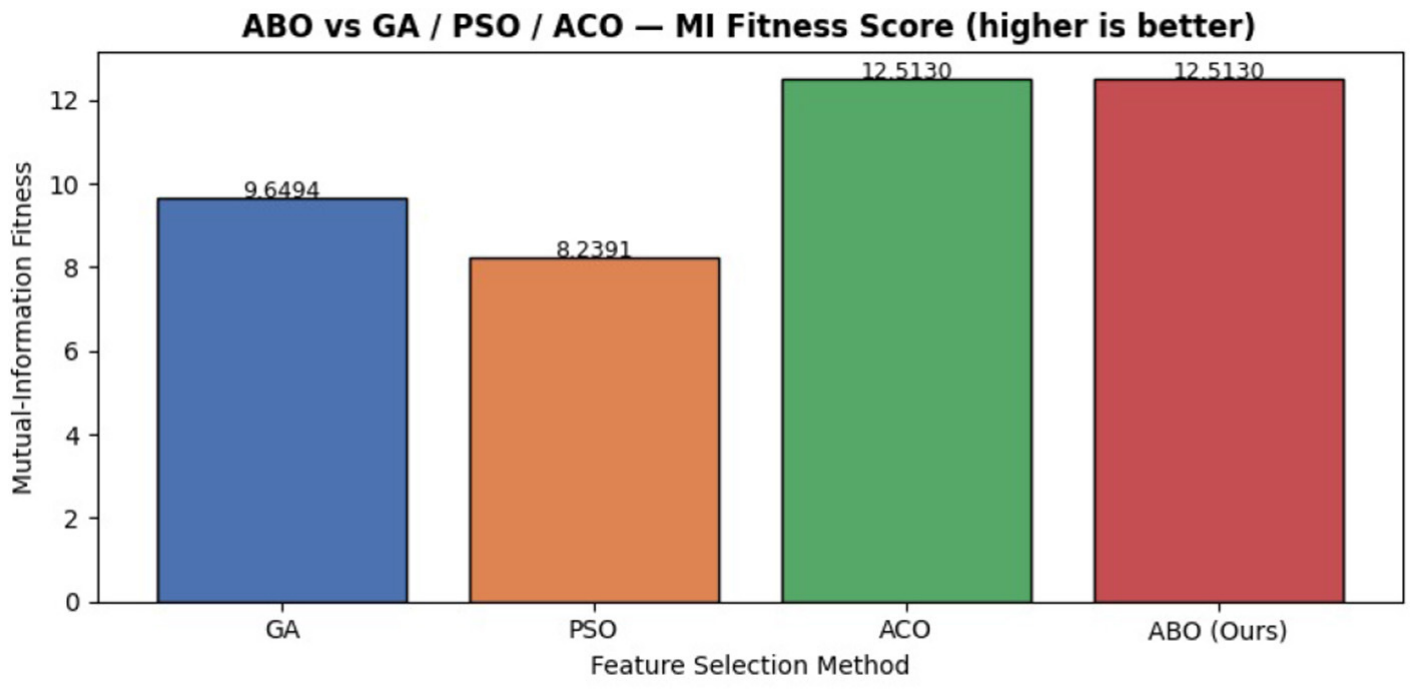
ABO vs. alternative bio-inspired feature selectors.

As shown in the Figure 20, the proposed ABO method achieved an MI score of 12.513 higher than GA with 9.649 and PSO with 8.239 and comparable to ACO with similar score. While the ACO demonstates competitive performance due to its pheromone-guided probabilistic selection mechanism, the proposed ABO achieves similar effectiveness through a more structured and deterministic search strategy. Specifically ABO employs an MI-greedy selection mechanism combined with deteministic swap mutation, to enable a balanced trade-off between exploration and exploitation without relying on stochastic pheromone updates.

In contrast, GA relies on binary-encoded crossover and mutation operations resulting in slow convergence and suboptimal feature subsets due to its randomness. Similarly, PSO features subset are updated based on the continuous velocity-position dynamics, a mechanism that is not naturally aligned with discrete feature selection problems and often results in lower quality solutions.

The most important observation is the consistency provided by the ABO. Even with similar score to ACO, ABO relies on a simpler and more computationally robust design in reducing the sensitivity to the parameter tuning and stochastic variablity. This makes ABO best suited for practical deployment in where compuational efficiency and reproducibilty are essential. On the whole, the effectiveness of the model is validated using the selected high-quality features achieved using the ABO algorithm thereby enhancing the overall model performance. The improved stability and reduced complexity supports the suitability of ABO as a robust feature selection method in the proposed intrusion detection framework.

### 6.5 Limitations

While the findings are significant, our research is limited in a number of ways that have to be taken into account. Primarily, the evaluation is somewhat constrained as it depends on a single, even sizable, dataset only. Because this limitation prohibits the generalizability of the findings. The reason for this is that the model may perform poorly with different and changing traffic patterns for other network environments that are still based on the real world. Secondly, although our model has shown an average good performance at the macro level, we discovered that its performance fluctuated for particular attack classes and it was still vulnerable to class imbalance and overlapping traffic characteristics. This vulnerability implies that just as some kinds of attacks are very faint, there can also be new ones which the detection system is unable to recognize.

Thirdly, the computational complexity which is intrinsic to BiLSTM architectures significantly hampers the possibilities of their being deployed in environments with limited resources such as IoT and edge devices where low-latency and real-time processing are of the highest importance.

Lastly, the platform functions in a static and autonomous manner and does not have an adaptive learning engine. As a result, they cannot independently refresh their knowledge base in order to be able to identify new or zero-day threats unless they go through very expensive retraining cycles. The stratified reduction of the CICIoT23 dataset works with around 1 million records may result with feasiblility in computations but may limit the exposure of some attack patterns and rare attacks. The diversity in the real-world attack behavior needs validation across several hetereogeous datasets. Finally, the use of recurrent architecture may limit deployment in resource contrained edge and IoT environments.

### 6.6 Future work

Future research will focus on several critical areas to not only extend this work but also overcome its limitations. We will verify the robustness and generalizability of the model in a rigorous manner by cross-dataset validation with several public benchmark datasets ensuring the diversity among the network environments. To make the device more deployable, we will study model optimization techniques such as pruning, quantization, and knowledge distillation to create a lightweight version that will be appropriate for edge computing. Besides that, it is our main intention to further the core architecture; we might substantially increase the ability of the model to discriminate features of complex attack classes by simply integrating attention mechanisms or by investigating hybrid models with Graph Neural Networks (GNNs).

The most important goal, probably, is to make a system that is able to learn from new data without forgetting the old one and hence can be updated continuously by online or incremental learning techniques; in this way, it will be possible for the model to develop together with the new threats. To build up confidence and facilitate the practical use of the model, we want to incorporate Explainable AI (XAI) techniques so that the reasoning of the model can be clear to security analysts. Finally, we will find ways to make the model less vulnerable to adversarial attacks so that it can be stable and reliable even when sophisticated evasion attempts are made.

### 6.7 Summary

The proposed BiLSTM-driven IDS has been able to classify various types of attacks efficiently, and this is well supported by high macro and weighted metrics of the evaluation. However, issues of reliance on the dataset, a high consumption of resources, and the ability to adjust to new threats still linger, thus, there is room for enhancement of the present solution. If these problems are solved by means of cross-dataset validation, model optimization, hybrid architectures, and adaptive learning mechanisms, it will result in an advanced IDS capable of delivering top performance and being operationally efficient in real-world scenarios.

## 7 Conclusion

The experimental results of the BiLSTM intrusion detection model proposed depict that hybrid recurrent-attention architectures can, after being combined with strong preprocessing, balanced data distribution and optimized hyperparameters, reach very high accuracies and be stable across different categories of attacks on IoT devices. The model thus obtained a test accuracy of 97.32%, a macro F1-score of 0.9732, and a Cohen's Kappa score of 0.9694 which is an indication of strong agreement and hence, reliability in deployment in real-life situations. The classification report emphasizes the stability of performance of the model for each of the classes, where in most of the attack classes, precision and recall are almost one. For example, BruteForce, Mirai, and Web have high precision and recall values which means that very few false negatives have been detected and the detection sensitivity is excellent. The slightly lower values in Benign and DDoS are due to some features being common in normal as well high-traffic patterns, however, the overall effect is still very small. Misclassification tables explain that misclassification is mostly between the groups with similar temporal signatures thus confirming that the model is able to capture the temporal dependencies from both directions efficiently.

ROC as well as Precision-Recall curves testify to the high discrimination power of the model with extremely small trade-offs between precision and recall. Accuracy per class against support further reveals that the system can maintain its performance at a very high level even when there is a class imbalance–this has been made possible by stratified sampling, synthetic oversampling (SMOTE), and adaptive loss optimization. Training convergence plots illustrate the model's stable learning process turning up no indications of overfitting, therefore, the model can generalize well to new traffic data.

On a real-time basis, the inference evaluation came up with an average prediction latency of 0.219 ms per sample and throughput of more than 4,500 samples/s, thus ensuring the real-time online intrusion detection practicality. The radar chart comparison is an indication of the equally good performance of precision, recall, and F1-score in all categories and thus the reliability of the architecture's holistic and stable behavior under various conditions. Different from standard ML and RNN-directional single methods, the proposed hybrid architecture makes use of bidirectional sequence modeling and multi-head attention to capture both local and global contextual relationships. Consequently, this interplay increases temporal interpretability, feature relevance, and robustness against noise in high-dimensional IoT data.

However, it is difficult to say that there are no limitations. There is a possibility that the overlapping traffic patterns of benign and spoofing may still cause a little ambiguity. In addition, the power consumption associated with multi-head attention and recurrent layers may limit the capacity of low-power edge devices on which the deployment will be carried out. On top of that, although the evaluation on CICIoT2023 can serve as evidence for good generalization, testing further on heterogeneous and adversarial datasets is necessary for a robustness certificate that is complete.

To put it briefly, the BiLSTM-based IDS provides the performance metrics of modern-day technology in terms of accuracy, latency, and throughput thereby positioning it as a suitable candidate for IoT security frameworks of the next generation. The upcoming work will take into account lightweight transformer variants, graph-based relational modeling, and continual learning mechanisms to guarantee intrusion detection that is adaptive, energy-saving, and zero-day-resilient. The present piece of work is an affirmation of the potential of temporal-attention hybrid deep models in terms of scalability and reliability as enablers for intelligent cybersecurity infrastructure.

## Data Availability

The original contributions presented in the study are included in the article/supplementary material, further inquiries can be directed to the corresponding author.
